# A hidden T-DNA-linked inversion-duplication is associated with a light-dependent *Arabidopsis* mutant phenotype

**DOI:** 10.1007/s00299-026-04030-1

**Published:** 2026-11-03

**Authors:** María del Pilar Martinez, Julie Anne V. S. de Oliveira, Ioana Nica, Noah Ditz, Ke Zheng, Vera Wewer, Sabine Metzger, Philipp Westhoff, Holger Eubel, Iris Finkemeier, Markus Schwarzländer, Boas Pucker, Veronica G. Maurino

**Affiliations:** 1 Molecular Plant Physiology, Institute for Cellular and Molecular Botany (IZMB), University of Bonn, Kirschallee 1, 53115 Bonn, Germany; 2 Plant Biotechnology and Bioinformatics, Institute for Cellular and Molecular Botany (IZMB), University of Bonn, Kirschallee 1, 53115 Bonn, Germany; 3 Institute of Plant Genetics, Leibniz University Hannover, Herrenhäuser Str. 2, 30419 Hannover, Germany; 4 Plant Energy Biology, Institute of Plant Biology and Biotechnology (IBBP), University of Münster, Schlossplatz 8, 48143 Münster, Germany; 5 Plant Metabolism and Metabolomics Facility, Institute for Plant Sciences, University of Cologne, and Cluster of Excellence on Plant Sciences (CEPLAS), Zülpicher Strasse 47B, 50674 Cologne, Germany; 6 Plant Metabolism and Metabolomic Facility, Düsseldorf and Cluster of Excellence on Plant Sciences (CEPLAS), Heinrich Heine University, Universitätsstraße 1, 40225 Düsseldorf, Germany; 7 Plant Physiology, Institute of Plant Biology and Biotechnology (IBBP), University of Münster, Schlossplatz 7, 48149 Münster, Germany

**Keywords:** Gene dosage, Genotype–phenotype association, Large scale chromosomal rearrangement, T-DNA insertion lines

## Abstract

**Key message:**

A hidden T-DNA-linked genomic rearrangement alters gene dosage and exacerbates light-dependent phenotypes in Arabidopsis, highlighting the importance of structural genome validation for reliable interpretation of mutantphenotypes.

**Abstract:**

T-DNA insertion mutants are widely used to study gene function, yet associated genomic alterations can confound phenotypic interpretation. We investigated mutants affecting mitochondrial malate dehydrogenase (MDH1) and heterodimeric NAD-dependent malic enzyme (ME1/ME2) under different photoperiods and irradiance regimes. Under long-day conditions, mutant combinations showed no major phenotypic differences from wild type. Under short-day, low-light conditions, *mdh1.1 me2* mutants were markedly smaller than wild type and, unexpectedly, more severely affected than *mdh1.1 me1 me2* triple mutants. ME1 was undetectable in *mdh1.1 me2*, indicating that both genotypes effectively lack heterodimeric NAD-ME. Whole-genome analysis identified a 137-kbp inverted duplication downstream of the MDH1 insertion in *mdh1.1 me2*, absent from the triple mutant. The original *mdh1.1* population segregated for the severe phenotype, whereas an independent *mdh1.2* allele did not reproduce it, linking the defect to the *mdh1.1*-associated rearrangement. The duplication increased gene dosage and transcript abundance, particularly under low light. Proteomics detected only five of 38 proteins encoded within the duplicated region, including phosphoenolpyruvate carboxylase 1 (PEPC1). *mdh1.1 me2* accumulated oxaloacetate-derived amino acids and exhibited altered carbon/nitrogen balance. These findings suggest that the duplication contributes to the phenotype, potentially through increased PEPC1 activity and consequent perturbation of carbon–nitrogen metabolism. However, interactions with MDH1 deficiency cannot be excluded, making PEPC1 a candidate contributor rather than the sole causal determinant. Our results highlight how T-DNA-linked structural variation can confound condition-dependent phenotypes and emphasize the need for structural validation of insertion loci, particularly given the widespread use of Agrobacterium-mediated DNA transfer in plant genome editing.

**Supplementary Information:**

The online version contains supplementary material available at https://doi.org/10.1007/s00299-026-04030-1.

## Introduction

*Agrobacterium tumefaciens* has been a cornerstone of plant genetic transformation for decades, enabling efficient delivery of DNA into the nuclear genome and thereby accelerating functional genomics in many species. In its natural infection process, *A. tumefaciens* transfers a defined segment of its tumor-inducing (Ti) plasmid, termed transferred DNA (T-DNA), into plant cells, where it may integrate into the host genome and drives crown gall formation (Van Larebeke et al. 1975; Chilton et al. 1977). T-DNA transfer relies on virulence (*vir*) genes located on the Ti plasmid, but outside the T-DNA region (Akiyoshi et al. 1984; Schröder et al. 1984; Zambryski et al. 1989). The T-DNA itself is delimited by imperfect ~25-bp border repeats (left and right borders; LB and RB) that are required for processing and integration. The internal native T-DNA sequence can be replaced by engineered cargo, while retaining the borders, which forms the very basis of routine plant transformation (Wang et al. 1984). The development of binary vector systems, in which the T-DNA and *vir* functions are provided on separate replicons, further simplified transformation workflows and enabled large-scale mutant generation (de Framond et al. 1983; Hoekema et al. 1983; Alonso et al. 2003; Lee and Gelvin 2008; Szarzanowicz et al. 2025).

Beyond transgenesis, T-DNA insertion mutagenesis has become a standard strategy to infer gene function by disrupting coding sequences or regulatory regions (Marks and Feldmann 1989; Koncz et al. 1990; Feldmann 1991). Following publication of the first *Arabidopsis thaliana* genome sequence (Arabidopsis Genome Initiative 2000), community resources expanded rapidly. Indexed insertion collections, together with searchable databases, enabled routine identification of presumed loss-of-function alleles (Meinke et al. 2003; Alonso and Ecker 2006). Today, hundreds of thousands of *A. thaliana* insertion lines are available, including major collections, such as SALK, GABI-Kat, SAIL, and WISC (Sussman et al. 2000; Sessions et al. 2002; Alonso et al. 2003; Rosso et al. 2003; Kleinboelting et al. 2012; O’Malley et al. 2015). This scale has made T-DNA lines indispensable for dissecting pathways ranging from metabolism to development and stress signaling.

Yet, the apparent genetic simplicity insertion of T-DNAs into plant genomes has limits that may be overlooked. While T-DNA integration is frequently described as largely random and stable (Thomas et al. 1994; Azpiroz-Leehan and Feldmann 1997; Gelvin 2017), its molecular route is closely tied to host DNA double-strand break repair. In practice, many insertion events are complex: truncated borders, tandem repeats, multiple T-DNA copies per genome (with an average of 1.5 T-DNA copy per T-DNA line) and the inclusion of unintended plasmid backbone sequence are common (Castle et al. 1993; Gelvin 2003, 2017; O’Malley et al. 2015; Nicolia et al. 2017; Pucker et al. 2021). Moreover, double-strand break repair itself can generate structural variants, via processes, such as single-strand annealing or non-allelic homologous recombination. In addition, T-DNA lines have repeatedly been found to carry large deletions, inversions, duplications, insertions, and translocations linked to the integration event (Nacry et al. 1998; Clark and Krysan 2010; Krispil et al. 2020). Such collateral changes can alter gene dosage or regulatory landscapes and may confound genotype-phenotype interpretation, especially when phenotypes are subtle, conditional, or interpreted through genetic comparisons (Tax and Vernon 2001; Valentine et al. 2012; Tamura et al. 2016). To characterize T-DNA insertion lines, several techniques can be used, ranging from PCR-based approaches to long-read genotyping (Liu et al. 1995; O’Malley et al. 2007; Głowacka et al. 2016; Pucker et al. 2021).

In *A. thaliana*, mitochondrial malate is metabolized primarily through mitochondrial malate dehydrogenase (MDH) and NAD-dependent malic enzyme (NAD-ME), two enzymatic systems that provide distinct, but interconnected routes for mitochondrial carbon metabolism. Of the two mitochondrial MDH isoforms, MDH1 is the predominant isoform in green tissues, showing substantially higher transcript and protein abundance than MDH2 (Tomaz et al. 2010; Hüdig et al. 2015; Fuchs et al. 2020). Loss of MDH1 causes a relatively mild growth phenotype under ambient conditions, but its impact becomes much more pronounced under low CO_2_, where *mdh1* plants show strongly reduced biomass and substantial perturbations of photorespiratory metabolism (Tomaz et al. 2010; Lindén et al. 2016). Biochemical characterization further showed that recombinant *A. thaliana* MDH1 predominantly occurs as a homodimer and that acetylation of conserved lysine residues fine-tunes its catalytic properties, providing an additional layer of regulation of mitochondrial malate metabolism (Balparda et al. 2022). *A. thaliana* NAD-ME is encoded by two genes, *ME1* and *ME2*, whose products assemble in vitro into enzymatically active homo- and heteromeric enzymes with distinct kinetic and regulatory properties (Tronconi et al. 2008, 2010). Plants lacking NAD-ME activity display no pronounced growth phenotype under standard autotrophic conditions, but show substantial alterations in nocturnal metabolism, particularly in TCA-cycle intermediates and associated amino acids (Tronconi et al. 2008).

Given the complementary roles of MDH1 and NAD-ME in mitochondrial malate metabolism, we sought to determine how their combined impairment affects plant development and physiological performance across different light environments. We, therefore, examined T-DNA insertion lines impaired in MDH1 (Tomaz et al. 2010; Sew et al. 2016) and in ME1 and ME2 (Tronconi et al. 2008). Unexpectedly, the *mdh1.1 me2* double mutant exhibited a much stronger light-dependent phenotype than the *mdh1.1 me1 me2* triple mutant, suggesting that the phenotype could not be explained solely by the intended disruption of mitochondrial malate metabolism and pointing to an additional layer of genetic complexity. Here, we shed light on the genetic basis of this observation by combining physiological phenotyping with whole-genome analysis. Our data illustrates how insertion-associated genome restructuring can influence mutant phenotypes. By linking a T-DNA-associated large-scale duplication to altered gene dosage and downstream molecular and physiological readouts, our study provides a compelling case for the need of caution when inferring functional effects based on T-DNA-insertions, validating genome structure around insertion alleles emerges as an important prerequisite for attribution of causality.

## Results

### Unexpectedly exacerbated phenotype of *mdh1.1 me2* relative to *mdh1.1 me1 me2* under short days

Recently, using T-DNA insertion mutants lacking the major mitochondrial malate dehydrogenase (MDH1) and the two subunits of NAD-malic enzyme (ME1 and ME2), we identified mitochondrial malate decarboxylation capacity as a key control point linking respiratory energy supply to plastid function under low-energy conditions (Martinez et al. 2026). Because NAD-ME acts predominantly as a heterodimer in vivo (Tronconi et al. 2008), we hypothesized that if leaf NAD-ME function depends on heterodimer assembly, then double mutants combining *mdh1.1* with loss of the single ME2 subunit (*mdh1.1 me2.1* and *mdh1.1 me2.2*) should phenocopy the *mdh1.1 me1 me2* triple mutant (Suppl. Fig. 1).

**Fig. 1:**
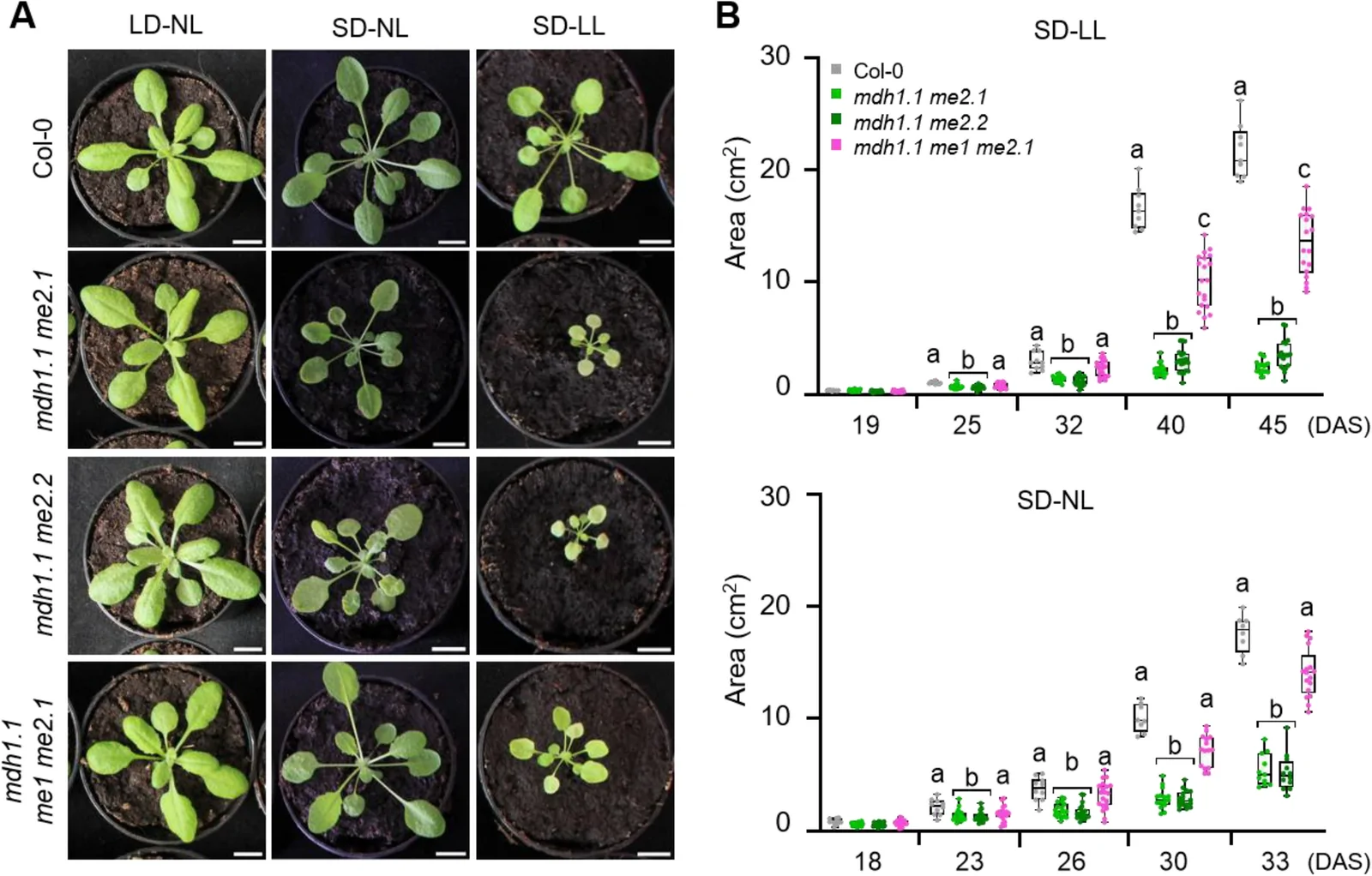
**Light-intensity- and photoperiod-dependent phenotypes of plants lacking MDH1 and ME.** **A** Representative plants of each genotype grown under long-day (LD) conditions at 120 µmol photons m^−2^ s^−1^ (NL) for 23 days, short-day (SD) at NL for 30 days, and SD at 60 µmol photons m^−2^ s^−1^ (LL) for 40 days. Scale bar, 1 cm. **B** Rosette area at different days after sowing (DAS) under SD at NL and LL (*n* = 10). Statistics were performed separately for each day using one-way ANOVA followed by Tukey’s post-hoc test. Different letters indicate statistically significant differences (*pval* < 0.05).

To test this hypothesis, we analyzed the growth of all genotypes under different photoperiods and light intensities. Under long-day conditions (LD) at 120 µmol photons m^−2^ s^−1^ (normal light, NL), no visible differences were observed among the genotypes (Fig. 1). In contrast, clear genotype-dependent differences emerged under short-day conditions (SD). Under SD-NL, the triple mutant was largely indistinguishable from the wild type, whereas both double mutants already exhibited reduced growth. When plants were exposed to the more energy-limiting conditions, SD at 60 µmol photons m^−2^ s^−1^ (low light, LL), these differences became substantially more pronounced. The double mutants displayed the most severe phenotype, characterized by pale-green leaves and a strong reduction in growth compared with the wild type (Fig. 1). Unexpectedly, this phenotype was considerably more severe than that of the triple mutant, which was also smaller than the wild type, but consistently larger and less chlorotic than either double mutant (Fig. 1). Moreover, the double mutants failed to flower under SD conditions.

### Low photosynthetic performance, thylakoid remodeling in *mdh1.1 me2*

**Fig. 2:**
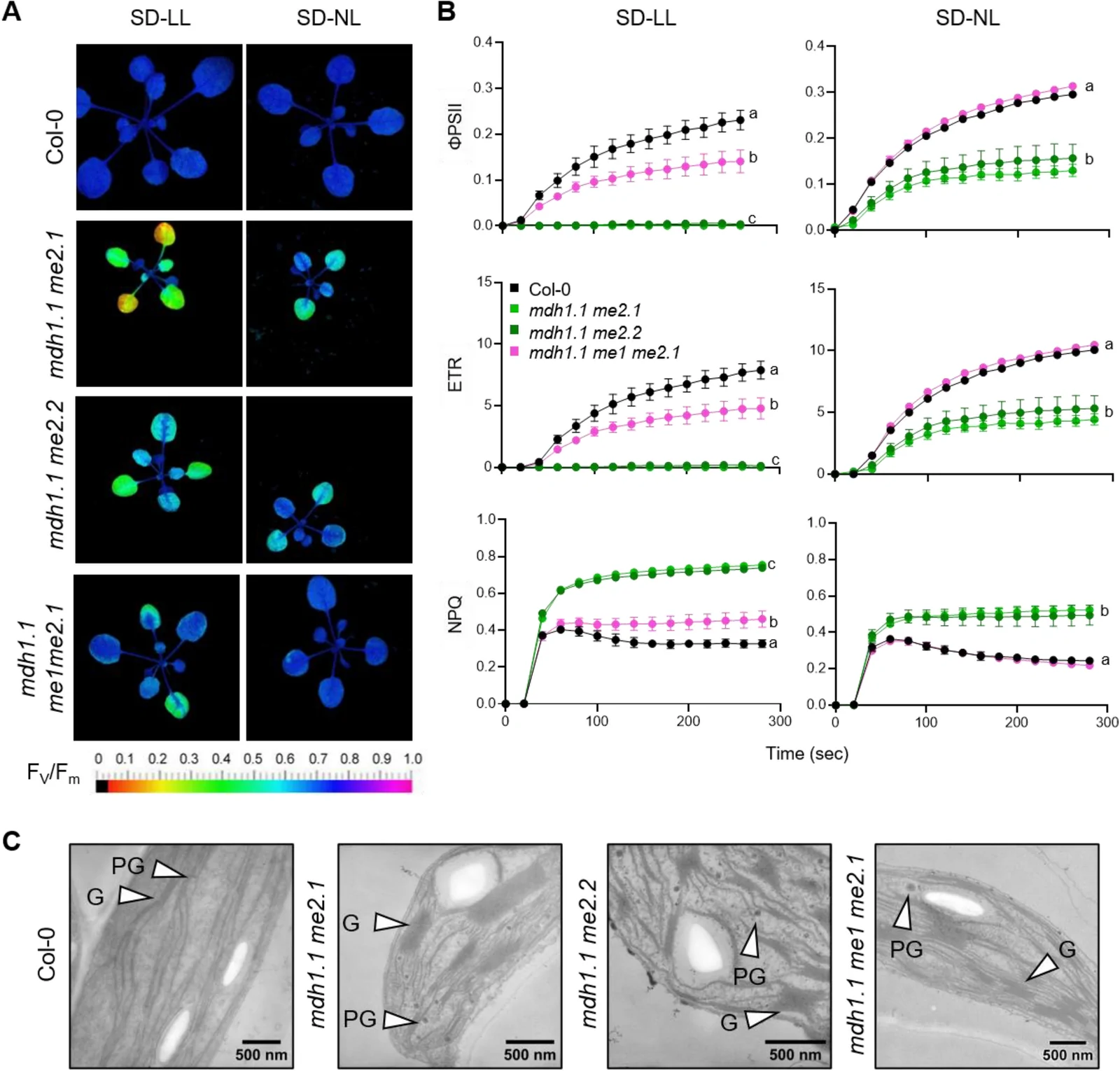
**Photosynthetic performance and chloroplast ultrastructure under SD.** **A** Representative false-color images of the maximum quantum yield of PSII (Fv/Fm). **B** Chlorophyll fluorescence parameters derived from imaging in (**A**): ΦPSII (effective quantum yield of PSII), ETR (electron transport rate), and NPQ (non-photochemical quenching). Values are means ± SE (*n* = 3). **C** Transmission electron micrographs of leaf sections from 40-day-old plants grown under SD at 60 µE and harvested at the end of the night. G, grana; PG, plastoglobuli. Data from *mdh1.1 me1 me2.1* were taken from Martinez et al. (2026). NL, normal light (120 µmol photons m^−2^ s^−1^); LL: low light (60 µmol photons m^−2^ s^−1^); LD, long-day; SD, short-day

Given the pale green phenotype, we further assessed the photosynthetic performance of plants grown under SD and either LL or NL conditions. Under both irradiance regimes, *mdh1.1 me2* exhibited a markedly lower photosynthetic capacity than Col-0 and the triple mutant (Fig. 2A and B). In LL, the maximum quantum efficiency of PSII (Fv/Fm) in *mdh1.1 me2* was reduced to ~50% of the value observed under NL. Consistent with strong impairment of PSII function, the effective quantum yield of PSII (ΦPSII) and the electron transport rate (ETR) were close to zero. This disruption also affected energy dissipation, as non-photochemical quenching (NPQ) increased and, in LL reached values ~2-times higher than in NL. In comparison, the triple mutant also showed reduced photosynthetic parameter values in LL than in NL, but these remained intermediate between those of wild type and double mutants. Under NL, the triple mutant showed wild-type-like values for all measured parameters.

Because *mdh1.1 me2* showed impaired photosynthetic performance, we next examined chloroplast ultrastructure. Under SD-LL conditions, chloroplasts from the double mutant showed marked grana hyperstacking, with thicker and more extensively stacked grana thylakoids, and exhibited an increased abundance of plastoglobules compared with the wild type (Fig. 2C). Enhanced grana stacking is commonly associated with a shift toward higher PSII and LHCII content relative to PSI, and may, therefore, reflect changes in the composition of photosynthetic complexes and/or pigment complement (Olive and Vallon 1991; Häusler et al. 2009; van Wijk and Kessler 2017). Increased plastoglobuli abundance is linked to leaf senescence and stress, consistent with a role for plastoglobuli in sequestering lipids and chlorophyll released during thylakoid remodeling (van Wijk and Kessler 2017; Zechmann 2019). A less pronounced increase in grana stacking was observed for *mdh1.1 me1 me2*, although without a detectable increase in plastoglobuli (Fig. 2C).

### ME1 activity is absent in *mdh1.1 me2*

The principal genetic difference between *mdh1.1 me2* and *mdh1.1 me1 me2* is the additional loss of ME1 in the triple mutant. To test whether differences in ME1 abundance or mitochondrial NAD-ME capacity are responsible for the stronger phenotype in SD, we measured NAD-ME activity in mitochondrial extracts, assessed ME1 protein levels by immunoblotting, and quantified protein abundance by LC–MS/MS-based proteomics.

**Fig. 3:**
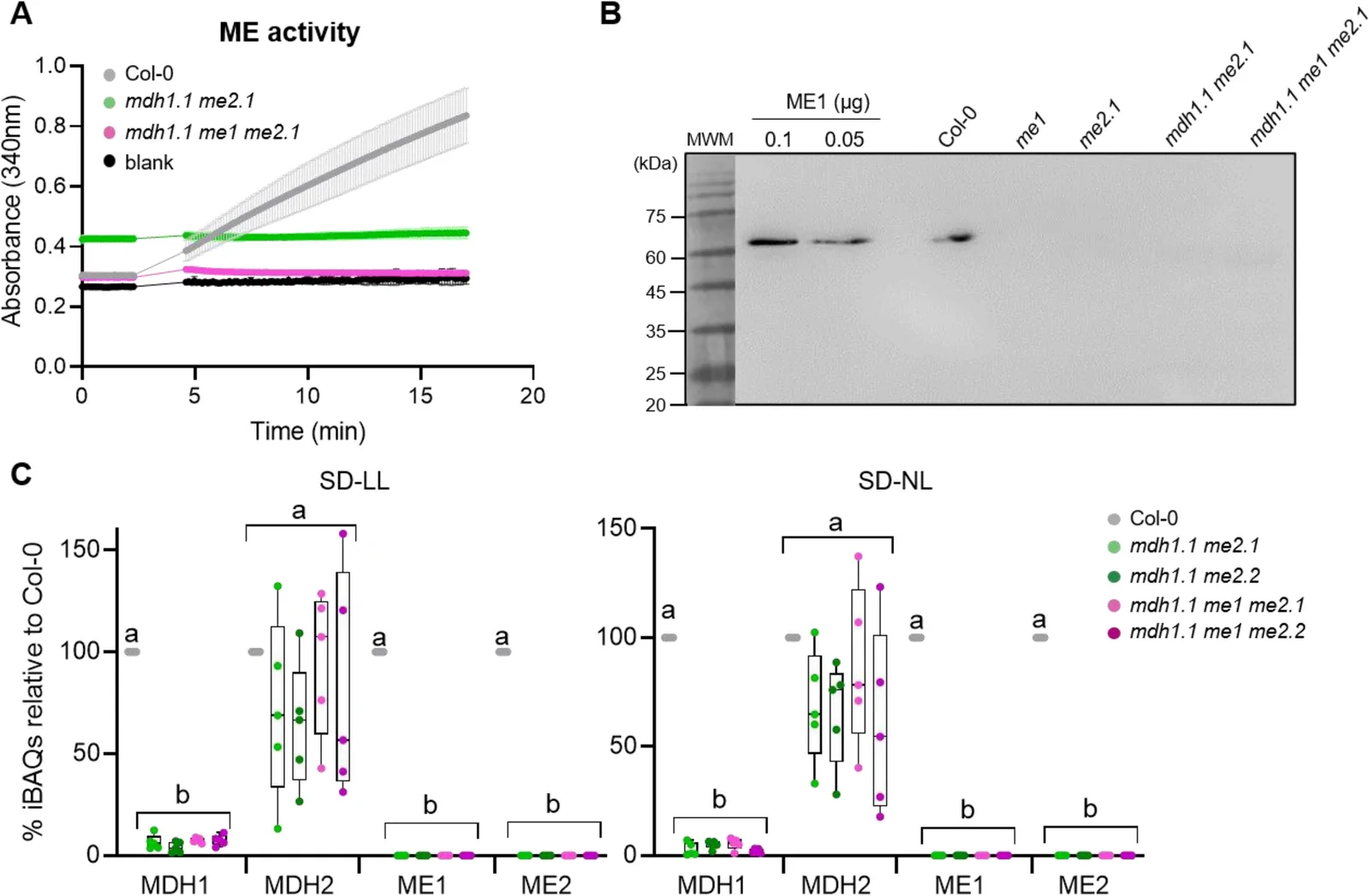
**Abundance and activity of mitochondrial ME and MDH in plants grown in SD.** **A** NAD-ME activity in mitochondrial extracts, quantified by monitoring NADH production at 340 nm. Data are means ± SD (n = 3). **B** Immunoblot of mitochondrial extracts from plants grown under SD at NL and harvested 1 h after lights-on (+ 1 h), probed with ME1-specific antibodies. 30 µg of mitochondrial proteins were loaded in each well and 7 µl of the MWM. MWM: molecular-weight marker (ROTI®Mark TRICOLOR (Carl Roth)). kDa: kilodaltons. **C** Percentage of Intensity-Based Absolute Quantification (iBAQs) relative to Col-0 of ME1, ME2, MDH1, and MDH2 proteins in plants grown under SD at low light (LL) or normal light (NL). Interleaved box and whiskers showing minimum to maximum values (*n* = 5). Statistics were performed separately for each day using one-way ANOVA followed by Tukey’s post-hoc test. Different letters indicate statistically significant differences (*pval* < 0.05). Proteomic data from *mdh1.1 me1 me2* was taken from Martinez et al. (2026)

In wild-type mitochondrial extracts, NAD-ME activity was readily detectable, whereas activity in leaves of both double and triple mutants was below the detection limit (Fig. 3A). Consistent with this, immunoblotting detected a clear ME1 band in wild type, but no immunoreactive signal in either mutant line (Fig. 3B). LC–MS/MS proteomics identified multiple ME1-, ME2-, and MDH1-unique-derived peptides in wild-type samples, whereas none of these peptides were detected in leaf extracts from either the double or triple mutant (Fig. 3C). In contrast, low levels of MDH2-derived peptides were detected at similar abundance in all genotypes. Together, the concordant absence of NAD-ME activity, ME1 immunoreactivity, and a lack of ME1 peptide detection indicate that ME1 does not accumulate in leaves of both mutant backgrounds. Accordingly, the enhanced phenotype of the double mutants is not the result of residual ME1 activity.

### Genome sequencing uncovers a major genomic rearrangement in *mdh1.1 me2*

**Fig. 4:**
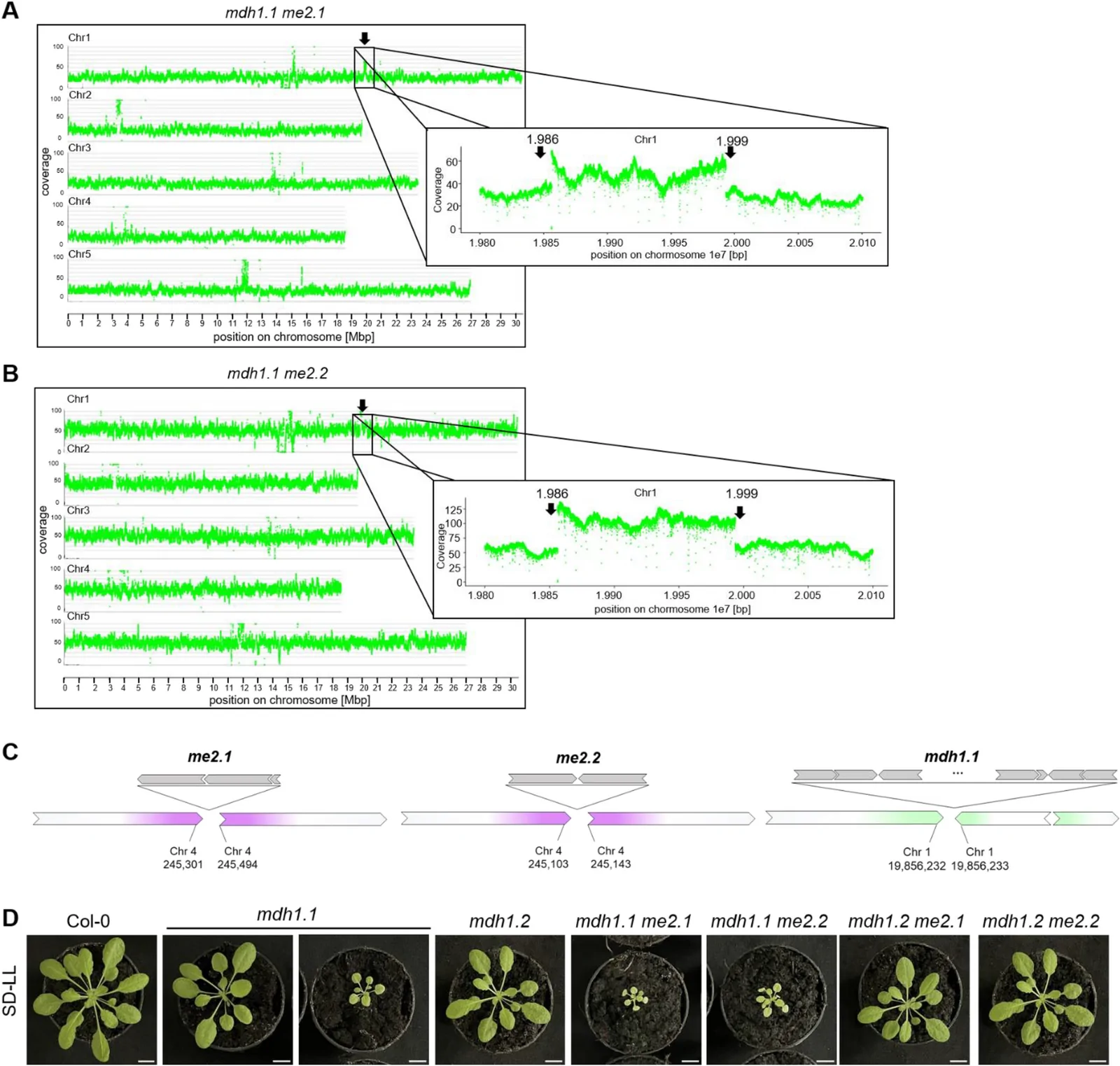
**An** ***MDH1*-linked inversion-duplication is associated with the small yellowish phenotype under SD-LL conditions.** **A, B** Read-depth coverage plots for (**A**) *mdh1.1 me2.1* and (**B**) *mdh1.1 me2.2*, generated with a custom Python script based on long read mappings (available at GitHub: https://github.com/bpucker/GKseq2). In both double mutants, a duplication was detected downstream of the T-DNA insertion site in *MDH1*. (**C**) Schematic overview of the chromosomes carrying the T-DNA insertions. T-DNA insertions are shown in gray, with arrowheads indicating insertion orientation. Segment lengths are not drawn to scale. (**D**) Representative plants of wild-type (Col-0), *mdh1.1*, *mdh1.2, mdh1.1 me2.1*, *mdh1.1 me2.2*, *mdh1.2 me2.1* and *mdh1.2 me2.2* knock-out mutants grown under SD-LL for 40 days. Scale bar, 1 cm

As *mdh1.1 me2* lacks detectable ME1 protein, we expected this line to phenocopy the *mdh1.1 me1 me2* triple mutant. However, its unexpectedly stronger phenotype prompted us to investigate whether additional genomic lesions might contribute to the observed defects. We, therefore, performed whole-genome sequencing of both double mutants using Oxford Nanopore Technologies (ONT) long-read sequencing. Local assemblies confirmed the presence of T-DNA arrays at genomic loci, *At1g53240* (MDH1) and *At4g00570* (ME2) (Fig. 4C).

Because T-DNA integration can lead to large-scale genomic rearrangements (Pucker et al. 2021), we analyzed the ONT sequencing data from *mdh1.1 me2.1* and *mdh1.1 me2.2* for structural variants. This analysis identified a major rearrangement at the *MDH1* locus in both double-mutant lines. In each case, an approximately 137-kb region immediately downstream of the *MDH1* T-DNA insertion site was duplicated. The duplicated segment ranges from position ~1.986 × 10⁷ to position ~1.999 × 10⁷ bp on chromosome 1 of the TAIR10 reference genome sequence and is inserted in an inverted orientation relative to the reference sequence (Fig. 4A-C). This interval includes 38 annotated genes (Fig. 4). Notably, the same duplication was not detected in the *mdh1.1 me1 me2.1* triple mutant (Martinez et al. 2026), indicating that the double- and triple-mutant lines differ in the presence of this *MDH1*-linked structural variant.

**Fig. 5:**
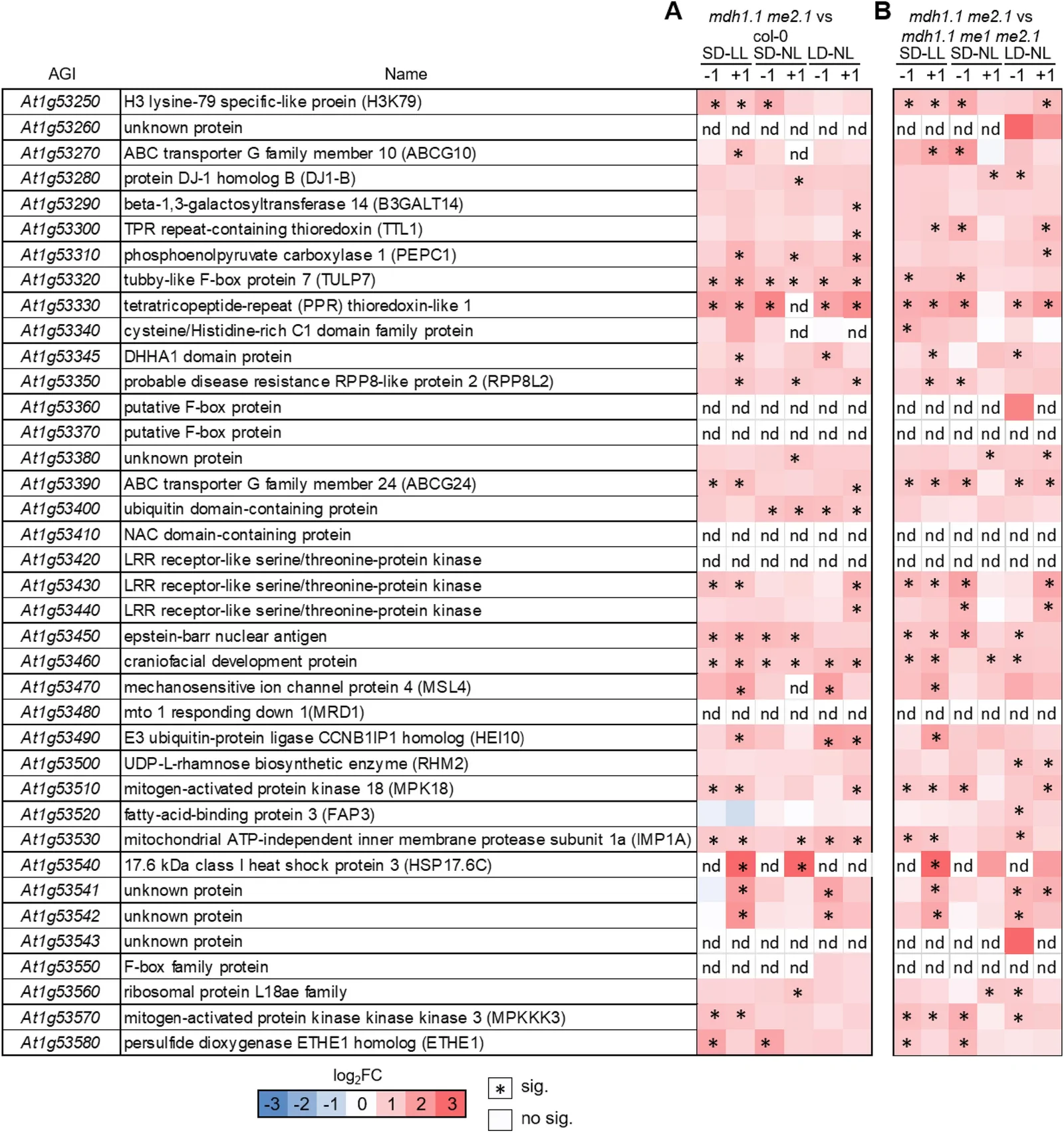
**Differential transcript abundance within the duplicated region in** ***mdh1.1 me2.1.*** Gene expression was quantified by RNA-seq in plants grown under short days (SD) at low light (LL) or normal light (NL) and under long days (LD) under NL. Samples were harvested 1 h before (− 1 h) and 1 h after (+ 1 h) dawn. Differential expression was summarized as log_2_ fold-change (log_2_FC) relative to (**A**) Col-0 and to (**B**) *mdh1.1 me1 me2.1*. Transcripts were classified as significantly changed when |log_2_FC|> 1 and *padj* < 0.05. nd, not detected; sig, significant; AGI: Arabidopsis Genome Initiative

Because the duplicated segment was located immediately downstream of the *MDH1* T-DNA insertion, we next examined the original *mdh1.1* single-mutant population under different photoperiods and light intensities. Under SD-LL conditions, *mdh1.1* segregated into distinct phenotypic classes, including wild-type-like plants and small, yellowish individuals (Fig. 4D), supporting an association between the *mdh1.1*-linked structural variant and the severe growth phenotype. To determine whether this phenotype was specific to the *mdh1.1* allele, we analyzed an independent *MDH1* insertion line, *mdh1.2*. In contrast to *mdh1.1*, *mdh1.2* plants displayed a uniform wild-type-like phenotype, and the corresponding *mdh1.2 me2* double mutant did not reproduce the severe growth defect observed in the *mdh1.1 me2* lines.

Together, these observations indicate that the exacerbated phenotype of *mdh1.1 me2* is most consistently associated with the *mdh1.1*-linked inversion-duplication and not with the combined loss of MDH1 and NAD-ME function itself. However, an interaction between duplication-associated changes and the loss of MDH1 may contribute to the severity of the phenotype.

These data provided the basis for reconstructing the most likely genetic scenario by which the duplicated segment was retained in the *mdh1.1 me2* mutants, but was absent from the *mdh1.1 me1 me2* mutants during line generation (Suppl. Fig. 2). The original *mdh1.1* parental population likely contained different *MDH1*-linked alleles carrying the same T-DNA insertion, but differing in the presence or absence of the adjacent inverted duplication. Importantly, mutant selection was performed under LD-NL conditions, under which no visible phenotype is apparent. Consequently, plants carrying or lacking the duplicated segment could not be distinguished phenotypically and were selected solely based on the presence of the desired T-DNA insertions. Thus, the *MDH1*-linked allele carrying the duplicated segment was selected by chance during the generation of the *mdh1.1 me2* double mutants, giving rise to plants with the enhanced light-dependent phenotype in SD. By contrast, an *MDH1*-linked allele lacking the duplicated segment was selected by chance during the generation of the triple mutant, resulting in plants that carry the intended T-DNA insertions, but lack the duplication. Together, this scenario explains how genetically related mutant lines acquired different structural configurations at the *MDH1* locus, leading to distinct phenotypic outcomes under changing conditions.

### The duplicated region is associated with light-regime-dependent changes in transcript abundance

Following identification of the genomic rearrangement, we assessed whether the duplicated interval was associated with altered transcript abundance under different light regimes using RNA-seq. Samples were collected from plants grown under SD conditions at LL and NL, and under LD conditions at NL, at one hour before dawn (− 1 h) and one hour after illumination (+ 1 h). Differential expression was calculated relative to Col-0 and visualized as log_2_ fold-change (log_2_FC) in a heatmap (Fig. 5A).

Within the duplicated interval, spanning *At1g53250-At1g53580*, transcripts from 32 genes were detected under all analyzed conditions. The strongest transcriptional differences between the duplication-carrying double mutant and Col-0 were observed at +1 h under SD-LL, when 19 of the 32 genes showed significantly higher transcript abundance (log_2_FC > 1; padj < 0.05). Under SD-NL, this effect was less pronounced, with 11 genes showing increased transcript abundance at the same time point. At − 1 h, 11 genes were already significantly elevated under SD-LL, compared with 7 under SD-NL (Fig. 5A; Suppl. Data 1).

Thus, although the duplication is a constitutive structural variant, its transcriptional consequences depend on the light regime and time of sampling. The strongest increase in transcript abundance within the duplicated interval occurred around the dark-to-light transition under SD-LL, suggesting that gene dosage associated with the duplication interacts with the prevailing light regime to produce condition-dependent transcriptional misregulation. This pattern is consistent with the pronounced phenotype of the duplication-carrying double mutant under energy-limiting SD-LL conditions.

To identify genes uniquely or commonly differentially expressed before (− 1 h) and after (+ 1 h) light onset under SD in LL and NL, we generated a Venn diagram (Suppl. Fig. 3; Suppl. Data 1). Below, we only focus on differentially expressed genes with functional annotation.

*Genes commonly accumulated under SD at LL and NL:* Three transcripts accumulated across all four conditions (SD-LL − 1/+1 and SD-NL − 1/+1): *At1g53320*, *At1g53450*, and *At1g53460*. *At1g53320* encodes the F-box protein, TULP7, a component of SCF-type E3 ubiquitin ligase complexes involved in proteasome-mediated protein turnover (Gagne et al. 2002; Bashore et al. 2023). Their consistent accumulation suggests a baseline regulatory response around the dark–light transition independent of light intensity. At − 1 h, *ETHE1* (*At1g53580*), encoding a mitochondrial sulfur dioxygenase involved in sulfur metabolism (Holdorf et al. 2012), accumulated under both LL and NL. After illumination (+ 1 h), three transcripts were commonly more abundant under both light intensities: *PEPC1* (*At1g53310*), *RPP8L2* (*At1g53350*), and *HSP17.6C* (*At1g53540*). Upon illumination, *HSP17.6C*, which encodes a small heat-shock protein, was strongly induced (~ 32-times under LL and ~16-times under NL), while *PEPC1*, encoding the anaplerotic enzyme phosphoenolpyruvate carboxylase 1, showed an increase of approximately threefold under LL and twofold under NL. These results indicate that the night-to-light transition activates metabolic and protein protection mechanisms largely independent of irradiance.

*Genes specifically accumulated under SD-LL:* Several transcripts accumulated preferentially under SD-LL. Four genes showed increased abundance at both time points: *ABCG24* (*At1g53390*), encoding a ABC transporter which are integral membrane protein complexes that use the energy of ATP hydrolysis to transport substrates across membranes; an LRR receptor-like kinase gene (*At1g53430*), predicted to encode a receptor involved in extracellular signal perception; *MAPK18* (*At1g53510*); and *MAPKKK3* (*At1g53570*), both encoding components of MAP kinase signaling pathways. Additional transcript abundances increased after illumination (+ 1 h), including *ABCG10* (*At1g53270*), predicted to encode an ABC transporter; *MSL4* (*At1g53470*), encoding a mechanosensitive ion channel; and *HEI10* (*At1g53490*), encoding an E3 ubiquitin ligase required for meiosis and overlapping with *MDR1*, a salicylic-acid-responsive gene. Together, these results suggest that higher irradiance preferentially enhances signaling-related responses, including membrane transport and MAP kinase pathways.

*Genes specifically accumulated under SD-NL:* Under SD-NL, *At1g53400*, encoding a ubiquitin-domain protein and putative regulator of ubiquitin-dependent protein degradation, accumulated at both − 1 and +1 h. After illumination (+ 1 h), *At1g53280*, encoding DJ1 homolog B (DJ1B) predicted to function in oxidative stress protection, and *At1g53560*, encoding a structural ribosomal protein of the L18ae family, accumulated specifically under NL conditions. These responses suggest that lower irradiance favors cellular maintenance processes, including redox protection, proteostasis, and translational capacity.

Comparison of gene expression within the duplicated region between *mdh1.1 me2.1* and *mdh1.1 me1 me2.1* showed that, overall, genes across this interval were more highly expressed in the double mutant, where the duplication is present, than in the triple mutant, where the duplication was not detected (Fig. 5B). This difference was most pronounced under LL conditions one hour after light onset (+ 1), when the highest number of differentially expressed genes was detected. At this time point, 17 of the 32 detected genes in the duplicated region were significantly upregulated in *mdh1.1 me2.1* compared with *mdh1.1 me1 me2.1*.

Together, these findings indicate that the duplicated genomic region integrates light-dependent transcriptional responses with pathways involved in metabolism, protein turnover, and stress signaling. While some genes respond similarly to the dark-light transition regardless of irradiance, lower irradiance (LL) primarily activates signaling pathways, whereas normal irradiance (NL) favors maintenance functions related to redox balance and protein homeostasis.

### Transcriptomic changes under SD-LL indicate altered hormone signaling, stress responses, and photosynthetic gene expression

To assess whether the duplicated region is associated with broader transcriptional changes, we compared transcript abundance between *mdh1.1 me2.1* and *mdh1.1 me1 me2.1* under SD-LL and SD-NL conditions, one hour before light onset (− 1) and one hour after light onset (+ 1). The strongest transcriptional differences were observed under SD-LL, particularly shortly after the transition to light (+ 1) and in SD-NL one hour before the light is on (− 1).

At +1 under SD-LL, 880 genes were expressed at lower levels and 595 genes at higher levels in *mdh1.1 me2.1* compared with *mdh1.1 me1 me2.1* (Suppl. Fig. 4A). Genes with reduced expression in *mdh1.1 me2.1* were mainly enriched in hormone- and growth-related processes, including auxin-related signaling and growth regulation (Suppl. Fig. 4B, Suppl. Data 2). In contrast, genes expressed at higher levels were enriched in mitochondrial RNA metabolic processes and RNA modification, as well as in responses to oxidative stress, cellular stress, and hypoxia (Supp. Fig. 3B, Suppl. Data 2). Notably, 19 of the 595 upregulated transcripts mapped to the duplicated region (Suppl. Data 1).

Transcriptional differences were less extensive at the end of the night under SD-LL. At − 1 under SD-LL, 95 transcripts were less abundant, and 152 transcripts were more abundant in *mdh1.1 me2.1* than in *mdh1.1 me1 me2.1* (Suppl. Fig. 4A). Among the genes expressed at lower levels in *mdh1.1 me2.1*, six were related to photosynthesis, including four chloroplast-encoded genes for photosystem I and photosystem II reaction center proteins (Suppl. Data 2). Among the genes expressed at higher levels, 12 mapped to the duplicated region (Suppl. Data 1). GO enrichment analysis of these higher expressed transcripts revealed a strong overrepresentation of stress-related categories (Suppl. Data 2).

In contrast, under SD-NL conditions, the largest transcript abundance changes occurred at the end of the night period (− 1). Compared with *mdh1.1 me1 me2.1*, *mdh1.1 me2.1* exhibited 1010 downregulated and 689 upregulated DEGs (Suppl. Fig. 4A). In contrast, shortly after the onset of the day (+ 1), only minor transcriptomic changes were detected, with 86 transcripts showing lower abundance and 79 transcripts showing higher abundance in *mdh1.1 me2.1* relative to *mdh1.1 me1 me2.1* (Suppl. Fig. 4A). At the end of the night period (− 1), KEGG pathway analysis revealed that transcripts associated with plant hormone signal transduction (ath04075) were significantly less abundant in *mdh1.1 me2.1*, whereas transcripts involved in the RNA transport pathway (ath03013) were significantly more abundant compared with *mdh1.1 me1 me2.1*. These results indicate that gene expression changes associated with light transitions are strongly influenced by light intensity.

Together, these results indicate that the duplicated region is transcriptionally responsive to the dark-to-light transition under SD-LL. Its presence is also associated with broader transcriptomic changes outside the duplicated interval, particularly affecting stress responses, hormone signaling, growth regulation, and photosynthesis-related gene expression.

### PEPC1 as a potential contributor to the enhanced *mdh1.1 me2* phenotype in SD

**Fig. 6:**
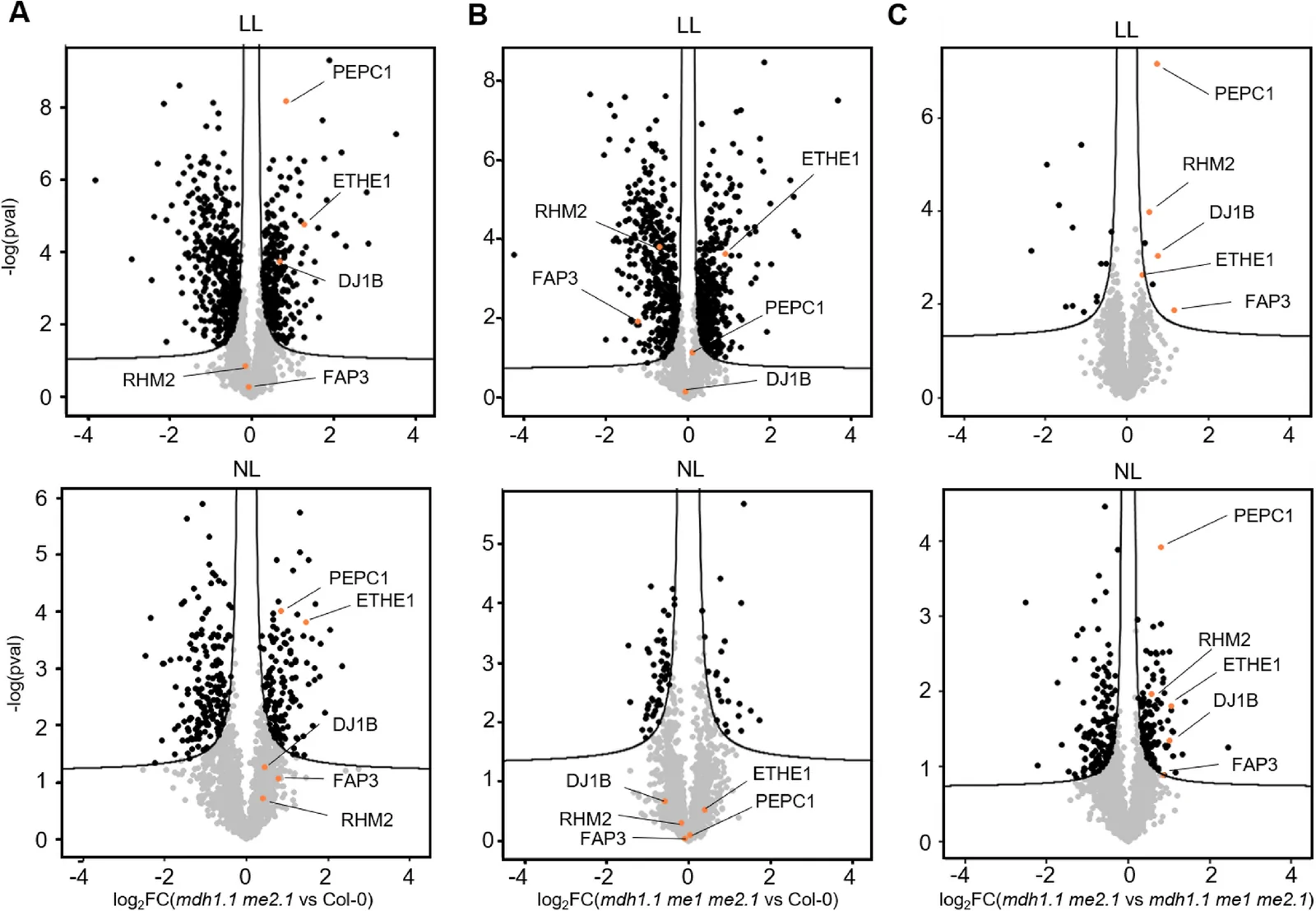
**Light-dependent abundance changes in protein groups between** ***mdh1.1 me2.1, mdh1.1 me1 me2.1*** **and Col-0 grown under LL and NL in SD.** Comparison of log_2_‐transformed LFQ‐values of (**A**) *mdh1.1 me2.1* and Col-0, (**B**) *mdh1.1 me1 me2.1* and Col-0 and, (**C**) *mdh1.1 me2.1* and *mdh1.1 me1 me2.1* protein groups grown in SD at LL or NL employing five independent biological replicates (Suppl. Data 3). Protein groups were graphed by fold change (log_2_FC; x-axis) and the confidence statistic (− log_10_(pvalue); y-axis). Orange points represent the proteins codified by the duplicated region. Protein groups located in the areas above the black lines fulfil the significance threshold limits (FDR ≤ 0.05; S_0_ = 0.1). Data from *mdh1.1 me1 me2.1* was taken from Martinez et al., (2026). ETHE1: ethylmalonic encephalopathy protein1; DJ1B: DJ-1 homolog B; FAP3: fatty-acid-binding protein 3; PEPC1: phosphoenolpyruvate carboxylase 1; RHM2: UDP-L-rhamnose biosynthetic enzyme; LL: low light; NL: normal light; SD: short day

To assess whether the duplicated interval was associated with altered protein abundance, we performed proteomic analysis of plants grown under SD-LL and SD-NL and harvested 1.5 h after lights-on. Of the 38 genes encompassed by the duplicated interval, only five corresponding proteins were detected: PEPC1, ETHE1, DJ1B, RHM2 (UDP-L-rhamnose biosynthetic enzyme), and FAP3 (fatty-acid-binding protein 3). RHM2 and FAP3 did not show increased accumulation in any of the conditions analyzed (Fig. 6A; Suppl. Data 3). Under LL, PEPC1, ETHE1, and DJ1B accumulated to higher levels in *mdh1.1 me2* than in Col-0, whereas under NL only PEPC1 and ETHE1 remained significantly increased (Fig. 6A; Suppl. Data 3). By contrast, in *mdh1.1 me1 me2*, only ETHE1 was more abundant under LL, while none of the remaining proteins showed significant increases under either light regime (Fig. 6B; Suppl. Data 3). Accordingly, the increased protein abundance observed in *mdh1.1 me2* is not detected in the triple mutant background lacking the duplicated genomic segment (Fig. 6C).

**Fig. 7:**
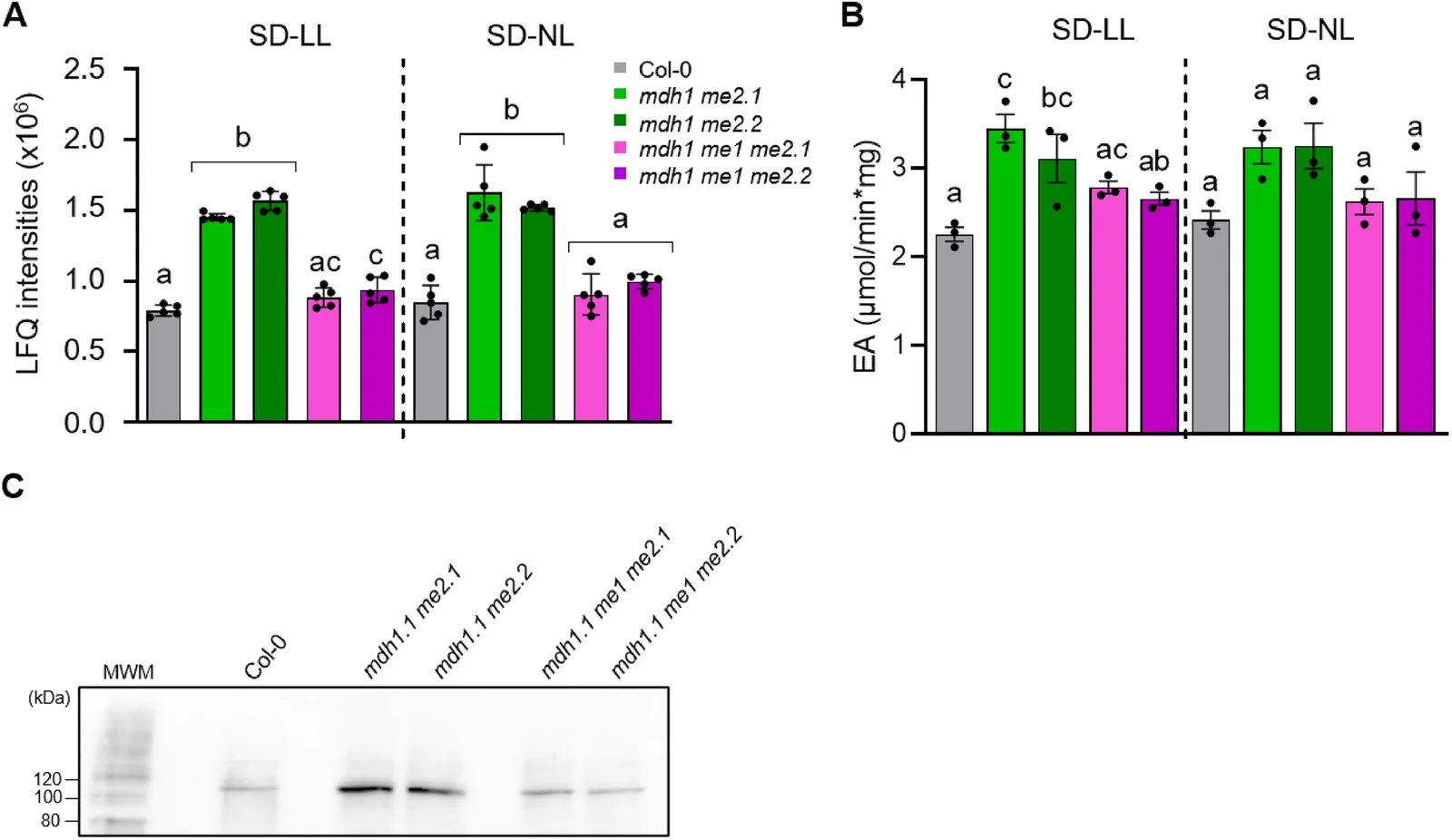
**Increased PEPC1 abundance and activity in** ***mdh1.1 me2. *** (**A**) Label-free quantification (LFQ) of PEPC1 protein abundance in plants grown under SD at LL or NL. Bars show means ± SD (*n* = 5). Proteomic data from *mdh1.1 me1 me2.1* was re-analyzed from Martinez et al., (2026). **B)** PEPC enzymatic activity in total protein extracts from plants grown under SD at LL or NL, harvested 1 h after lights-on (+ 1 h). Panels (**A**) and (**B**) use the same color code. For (**A**) and (**B**), statistical analyses were performed separately for each light intensity using one-way ANOVA followed by Tukey’s post-hoc test; different letters indicate significant differences (*pval* < 0.05). **C** Immunoblot of total protein extracts from plants grown under SD at LL, harvested 1 h after lights-on (+ 1 h), probed with a PEPC1-specific antibody. 50 µg of total protein extract was loaded in each well and 7 µl of molecular weight marker (Thermo ScientificTM PageRuler prestainedTM). MWM: Molecular Weight Marker; kDa: kilodaltons

Among these proteins, PEPC1 showed a particularly consistent increase in *mdh1.1 me2*, whereas its abundance in *mdh1.1 me1 me2* remained close to wild-type levels (Fig. 7A). To independently validate the proteomic data, we measured PEPC activity in total protein extracts from plants grown under SD-LL or SD-NL and harvested 1.5 h after lights-on. PEPC activity was significantly higher in *mdh1.1 me2* than in Col-0 under SD-LL (Fig. 7B). We further assessed PEPC1 abundance by immunoblotting using an anti-PEPC1 antibody. A band of approximately 110 kDa, consistent with the expected molecular mass of PEPC1, showed greater intensity in *mdh1.1 me2* than in Col-0 and the *mdh1.1 me1 me2* triple mutant, whereas both triple-mutant lines displayed band intensities comparable to those of the wild type (Fig. 7C).

Because the transcriptional consequences of the duplicated interval were dependent on the light regime, we also examined *cis*-regulatory elements within the PEPC1 promoter region (2 kb upstream of the transcription start site) using PlantCARE (Lescot et al. 2002). Several elements associated with light-responsive transcription were identified (Suppl. Table 3), including the G-box and GT1-motif, both of which have been characterized in *Arabidopsis* (Hiratsuka et al. 1994; Leivar et al. 2009). The presence of these elements provides a possible regulatory basis for the condition-dependent changes in PEPC1 expression.

Overall, the increased PEPC1 transcript and protein abundance, together with elevated PEPC activity, identify PEPC1 as a plausible contributor to the stronger phenotype of the duplication-carrying *mdh1.1 me2* lines under SD-LL. However, because the duplicated interval contains multiple genes, these data do not establish PEPC1 as the sole cause of the phenotype.

### Low light promotes ammonium accumulation and shifts carbon/nitrogen balance in *mdh1.1 me2* mutants

Given that PEPC1 activity has been implicated in the coordination of carbon and nitrogen metabolism in leaves, which is essential for optimal plant growth and development (Shi et al. 2015; Fañanás‐Pueyo et al. 2025), we next assessed whether the exacerbated *mdh1.1 me2* phenotype was associated with altered elemental composition. We quantified total carbon (C) and nitrogen (N) in SD grown plants under LL and NL across the diel cycle. Under LL, the C/N ratio was consistently reduced in the double mutants, with the most pronounced differences detected at the end of the day (+ 7) and the end of the night (− 1) (Suppl. Fig. 5A). This shift reflected both a decrease in total C throughout the diel cycle and an increase in total N at the end of the day (Suppl. Fig. 5A). In contrast, under NL we observed no significant genotype-dependent changes in C/N ratio, total C, or total N (Suppl. Fig. 5B).

To further examine N status, we quantified leaf ammonium (NH_4_^+^). Consistent with the bulk elemental measurements, NH_4_^+^ levels were indistinguishable among genotypes under NL, whereas under LL the *mdh1.1 me2* double mutants accumulated significantly higher NH_4_^+^ (Suppl. Fig. 5A). Elevated NH_4_^+^ can restrict growth, impair chlorophyll accumulation, and induce photoprotective responses (Britto and Kronzucker 2002; Podgórska et al. 2020), which is consistent with the pale-leaf and high-NPQ phenotypes observed in the double mutants.

### Light-regime-dependent accumulation of OAA-derived amino acids in the double mutants

To extend our analysis of the altered C/N balance and NH_4_^+^ accumulation observed in the double mutants with elevated PEPC1 activity, we next examined broader effects on central metabolism by untargeted GC-MS profiling of leaf extracts from plants grown under SD-LL, SD-NL, or LD-NL conditions. Samples were collected at three diel time points: 1 h before dawn (− 1 h), 1 h after dawn (+ 1 h), and 1 h before the end of the photoperiod (+ 7/+15 h).

**Fig. 8:**
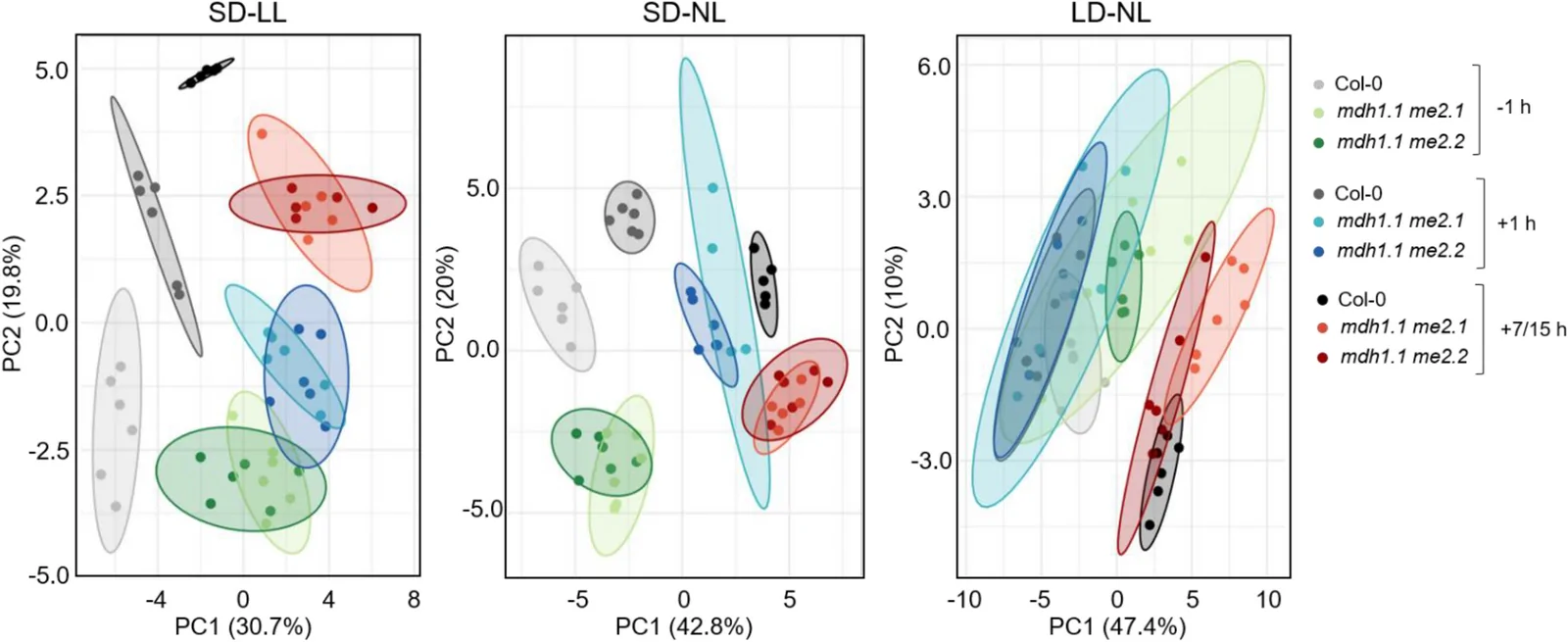
**Principal component analysis (PCA) of metabolic profile from** ***mdh1.1 me2***. Metabolites measured by GC-MS from whole rosette grown under SD-LL, SD-NL and LD-NL harvested in three time points: one hour before illumination (− 1 h), one hour after illumination (+ 1 h) and one hour before the end of the photoperiod (+ 7/15 h). Confidence ellipse of 80%. Each dot represents one biological replicate

Principal component analysis (PCA) showed that samples were separated primarily by growth regime and sampling time, while also revealing a clear genotype-dependent shift, particularly under low light (Fig. 8). Under SD-LL, both mutant lines, *mdh1.1 me2.1* and *mdh1.1 me2.2*, clustered distinctly from Col-0, indicating a pronounced alteration of the metabolic profile under low irradiance. Separation between the − 1 h and +1 h time points was additionally evident along PC2, consistent with a strong diel component. Under SD-NL, PC1 explained a larger fraction of the variance, and samples still showed genotype-associated clustering; however, the distance between *mdh1.1 me2* and Col-0 was reduced compared with SD-LL, suggesting partial normalization of the mutant metabolic profile under standard light. In LD-NL, sample separation was driven mainly by time after dawn, with the later time point (+ 15 h) displaced along PC1, whereas Col-0 and *mdh1.1 me2* overlapped more strongly at − 1 h and +1 h.

Taken together, the PCA indicates that SD-LL enhances the *mdh1.1 me2*-specific shift in metabolic composition, whereas under NL conditions the genotype effect is weaker and more strongly shaped by diel progression.

In plants grown under SD-LL, intermediates of the tricarboxylic acid (TCA) cycle and related metabolites were substantially altered (Suppl. Fig. 6 and 7). Malate accumulated in the double mutant at +1 h and +7 h, whereas pyruvate was consistently reduced across all time points. The pyruvate-derived amino acids: alanine, valine, and leucine increased at the end of the night (− 1 h). During the light period, valine remained elevated, while alanine declined significantly by +7 h, indicating differential regulation within this branch despite the overall reduction in pyruvate.

**Fig. 9:**
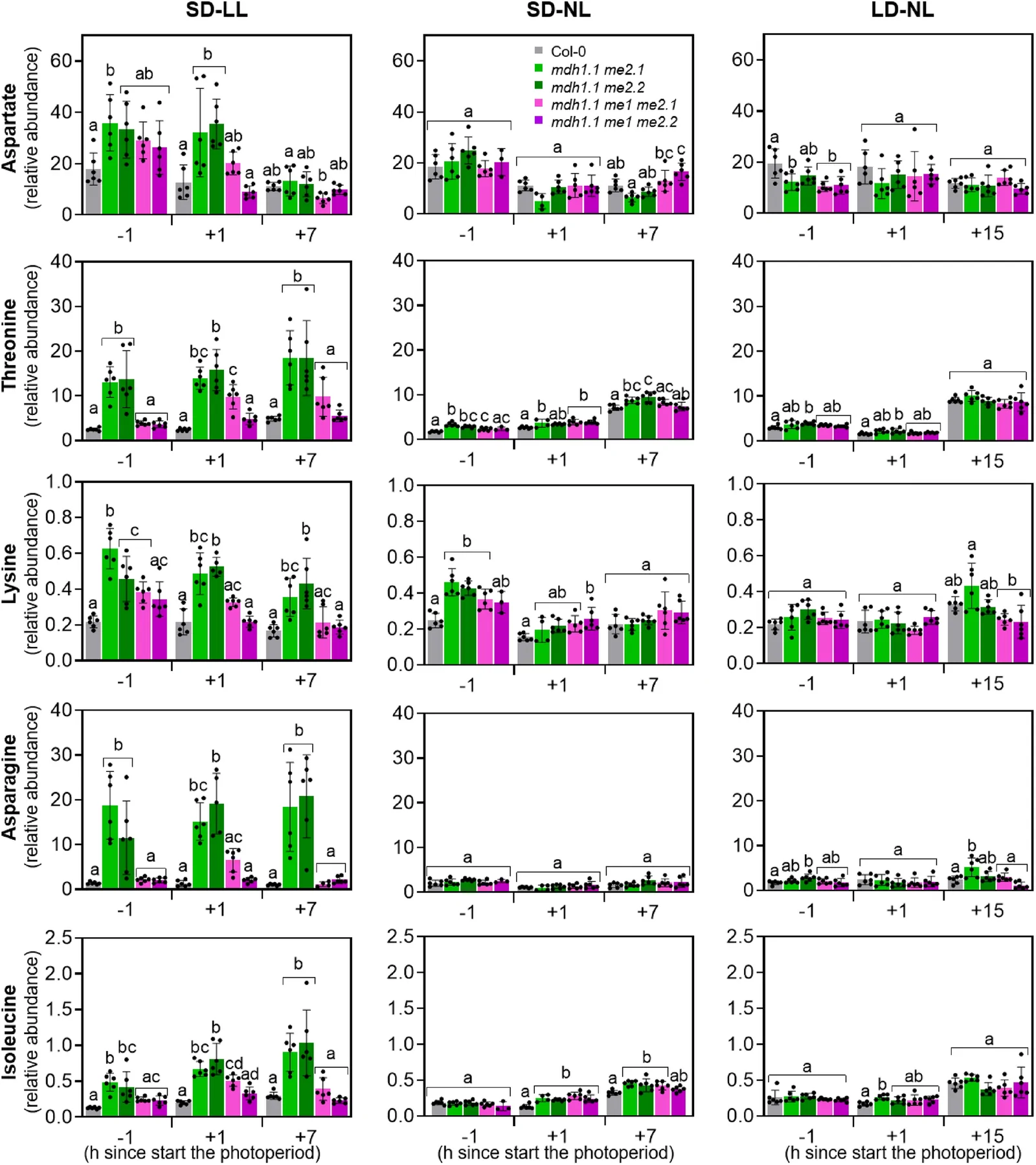
**OAA-derived amino acids levels in *mdh1.1 me2*** **and** ***mdh1.1 me1 me2.*** Relative metabolite abundances were determined by GC–MS in rosette leaves of the indicated genotypes grown under low light (LL) and normal light (NL) in short-day (SD) and long-day (LD) conditions. Samples were harvested 1 h before lights-on (− 1), 1 h after lights-on (+ 1), and 1 h before lights-off (+ 7 /+15). Bars represent means ± SD (*n* = 6 independent biological replicates). For each time point, statistical significance was assessed separately using one-way ANOVA followed by Tukey’s post-hoc test; significant differences relative to Col-0 (*pval* < 0.05) are indicated by asterisks. Data for the triple mutants were taken from Martinez et al. (2026)

GC-MS profiling further revealed a pronounced and coordinated accumulation of OAA-derived amino acids in the double mutant under SD-LL. Aspartate, lysine, threonine, isoleucine, and, most prominently, asparagine were elevated at all sampled time points (Fig. 9; Suppl. Fig. 6 and 7). While lysine, threonine, and isoleucine increased approximately fourfold relative to the wild type, asparagine showed an exceptional 15- to 20-fold increase. This pronounced accumulation of asparagine is consistent with enhanced N assimilation and storage, and aligns with the increased NH_4_⁺ levels and reduced C/N ratio observed under LL. Notably, this coordinated accumulation of OAA-derived amino acids was not detected in *mdh1.1 me1 me2* grown under the same conditions (Fig. 9; Suppl. Fig. 6 and 7), consistent with its association with the *mdh1.1*-linked duplication rather than with the combined impairment of MDH1 and NAD-ME function itself.

The concerted increase in aspartate-family amino acids suggests increased OAA availability and/or enhanced flux into OAA-dependent amino acid biosynthesis under LL. Given the increased PEPC1 abundance in the duplication-carrying *mdh1.1 me2* lines, one possible explanation is that enhanced PEPC-dependent anaplerosis increases cytosolic OAA supply, which under energy-limiting conditions may be preferentially directed toward N-rich end products (Suppl. Fig. 8). In this context, asparagine may serve as a major nitrogen sink when carbon availability is limiting. PEPC1 supplies OAA for several metabolic processes, including malate production and replenishment of TCA-cycle intermediates, while MDH1 contributes to mitochondrial malate/OAA interconversion; PEPC1 activity is also subject to feedback inhibition by malate (Krömer et al. 1996; Tcherkez et al. 2009; Abadie and Tcherkez 2019). Thus, increased PEPC1 abundance could, therefore, contribute to the altered carbon-nitrogen balance associated with the duplication directly or through interaction with the loss of MDH1 function.

Finally, the double mutants also showed increased accumulation of raffinose and proline (Suppl. Fig. 6 and 7), two metabolites commonly associated with low-light and carbon-limitation acclimation, as well as with stress-protective functions (Nishizawa-Yokoi et al. 2008; Szabados and Savouré 2010; Verslues and Sharma 2010; Jänkänpää et al. 2012). Their increase is consistent with a broader metabolic shift toward protective/maintenance states under LL, accompanying the diversion of C skeletons into N storage compounds.

### Phytohormone homeostasis is altered in *mdh1.1 me2* under SD

As an additional readout of the altered physiological state of *mdh1.1 me2*, we quantified phytohormones in plants grown under SD at LL and NL (Suppl. Fig. 9). The double mutant showed reduced levels of IAA (indole−3-acetic acid) and SA (salicylic acid) under both irradiance regimes, while JA (jasmonic acid)  accumulated specifically under LL. Cytokinin homeostasis was also altered, with reduced levels of trans-zeatin conjugates and increased accumulation of cis-zeatin-9-glucoside. These changes are consistent with the pronounced growth impairment, chloroplast remodeling, and metabolic perturbations observed in *mdh1.1 me2*. However, given the extensive alterations in these lines, it cannot be distinguished whether the changes in phytohormone homeostasis contribute to the phenotype or arise as secondary consequences.

## Conclusions and implications

### A linked genomic structural variant underlies the unexpected severity of the *mdh1.1 me2* phenotype

The severe, light-regime-dependent phenotype of the *mdh1.1 me2* double mutants cannot be explained solely by the combined loss of MDH1 and NAD-ME function, because the *mdh1.1 me1 me2* triple mutant displays a much milder phenotype despite similarly lacking detectable ME1 protein and NAD-ME activity. Thus, interpreting the phenotype solely on the basis of the intended MDH1 and ME2 mutations would be misleading.

Long-read whole-genome sequencing identified an approximately 137-kb inverted duplication immediately downstream of the *MDH1* T-DNA insertion in both *mdh1.1 me2* lines, irrespective of the *ME2* allele, whereas this structural variant was not detected in the *mdh1.1 me1 me2.1* triple mutant. Moreover, the original *mdh1.1* population segregates for the severe phenotype under SD-LL, whereas the independent *mdh1.2* allele does not reproduce this defect. Together, these observations strongly associate the exacerbated phenotype with the *mdh1.1*-linked inversion-duplication. The duplication may contribute substantially to the phenotype through altered dosage and expression of genes within the duplicated interval, while an interaction between duplication-associated changes and the loss of MDH1 cannot be ruled out.

### The *mdh1.1*-linked duplication alters gene expression and carbon–nitrogen metabolism

The duplicated interval is transcriptionally active rather than a silent structural variant. RNA-seq revealed increased transcript abundance across the duplicated region, with the strongest coordinated differences occurring around the dark-to-light transition under low irradiance.

Proteomic analysis detected only a subset of proteins encoded within the duplicated interval, among which PEPC1 showed the most consistent increase in abundance. This was supported by elevated PEPC activity, particularly under SD-LL conditions, and by immunoblot validation. In addition, several light-responsive cis-regulatory elements were identified in the *PEPC1* promoter, providing a possible basis for its condition-dependent expression. Together, these findings identify PEPC1 as a plausible link between the structural variant and the downstream metabolic alterations.

Consistent with increased PEPC1 abundance and activity, the *mdh1.1 me2* mutants displayed a pronounced shift in C/N balance under SD-LL, including ammonium accumulation and a coordinated increase in OAA-derived, aspartate-family amino acids, most notably asparagine. This metabolic signature is consistent with enhanced nitrogen assimilation and storage under carbon- and energy-limiting conditions. Thus, the available data support a model in which the *mdh1.1*-linked duplication contributes to the phenotype, potentially through increased PEPC1 abundance and activity and the resulting perturbation of carbon–nitrogen metabolism. However, the relative contributions of the duplication and the loss of MDH1 to these metabolic alterations cannot yet be disentangled, and an interaction between both factors remains possible.

PEPC1, however, may not be the sole contributor to the observed phenotype. The duplicated interval contains several additional genes involved in signaling, proteostasis, and stress responses that could influence growth, hormone homeostasis, chloroplast remodeling, or photosynthetic performance in a condition-dependent manner. Moreover, the inverted configuration of the duplicated segment and its breakpoint context may alter local regulatory architecture, such that expression changes do not necessarily follow a simple gene-dosage relationship. PEPC1 should, therefore, be considered a strong candidate contributor rather than the sole causal determinant. Distinguishing among these possibilities, and establishing a causal contribution of PEPC1, will require direct manipulation of PEPC1 dosage or activity in an appropriate genetic background.

### Future directions and implications for functional genomics

Disentangling loss-of-function effects from those associated with the duplication will require targeted genetic approaches. Key priorities include: i generating clean *MDH1* loss-of-function lines without associated structural rearrangements; ii genetically separating the duplication from the *mdh1.1* allele and establishing lines carrying the duplication in an otherwise Col-0 background; and iii testing whether increased PEPC1 dosage or activity is sufficient to reproduce key metabolic and phenotypic features in a clean genetic background. These approaches would not only establish the individual contributions of the duplication, MDH1 loss, and PEPC1 dosage, but also allow testing of potential genetic and metabolic interactions between PEPC1 and MDH1. Such interactions could be particularly relevant because increased PEPC-dependent OAA production may have different metabolic consequences when mitochondrial malate conversion is impaired. Resolving this relationship would provide broader insight into how anaplerotic carbon fixation and mitochondrial malate metabolism are coordinated to maintain carbon–nitrogen balance under energy-limiting conditions.

More broadly, our findings highlight an important consideration for plant functional genomics. When mutant phenotypes deviate from genetic expectations, particularly in an environment-dependent manner, structural characterization of insertion alleles becomes important for establishing genotype–phenotype relationships. T-DNA integration can be accompanied by extensive genomic rearrangements that complicate interpretation of mutant phenotypes. Long-read sequencing of *Arabidopsis* T-DNA insertion lines has revealed chromosome fusions, translocations, and large inverted duplications (Pucker et al. 2021). More recently, Gao et al. (2024) identified a T-DNA-associated megabase-scale inversion accompanied by translocation and duplication of a large chromosomal segment, resulting in altered gene expression and pleiotropic developmental phenotypes. Similarly, Tan et al. (2025) demonstrated that a phenotype initially attributed to loss of *SMO2-2* was instead caused by a T-DNA-induced reciprocal chromosomal translocation. Together with our findings, these studies emphasize that unexpected phenotypes in T-DNA insertion mutants may arise from complex structural alterations associated with the insertion event rather than solely from disruption of the annotated target gene. In such cases, long-read genotyping or sequencing provides a powerful means of resolving the underlying genomic structure and avoiding misinterpretation of mutant phenotypes (Pucker et al. 2021).

## Materials and Methods

### Plant material

The plant lines used in this study include *Arabidopsis thaliana* L. Heynh. ecotype Columbia (Col-0, wild type), along with homozygous T-DNA insertion lines Sail-291-C05 (*me2.1*), SALK_131720 (*me2.2*) and Sail-374-A02 (*me1*) (Tronconi et al. 2008) obtained from Nottingham Arabidopsis Stock Center (http://www.arabidopsis.info/) and, GK-097C10 (*mdh1.1*) and GABI_540F04 (*mdh1.2*) obtained from the GABI-Kat population (Rosso et al. 2003; Tomaz et al. 2010) (Suppl. Table 1). The T-DNA insertion sites were verified by sequencing using specific primers (Suppl. Table 2; Suppl. Fig. 1A). The *mdh1.1 me2* double mutants were generated by crossing the homozygous *mdh1.1* T-DNA insertion line with homozygous *me2.1* or *me2.2* plants. F1 plants were self-pollinated, and F2 progeny were screened by PCR using gene-specific primers in combination with T-DNA left-border primers to identify plants homozygous for both insertions (Suppl. Table 2). The *mdh1.1 me1 me2.1* triple mutant was generated by crossing the *mdh1.1 me2* double mutants with the *me1 me2* double mutant (Suppl. Fig. 2). The resulting progeny were selfed, and plants homozygous for the *mdh1.1*, *me1*, and *me2* T-DNA insertions were identified by PCR-based genotyping using specific primers (Suppl. Table 2). All mutant selection was performed under long-day conditions at normal light intensity.

### Plant growth

Plants were grown in soil (Floradur ® Substrate Multiplication Seed B, Rüttger Abstohs Gärtner-Einkauf GmbH&Co.KG, Siegburg, Germany) at 22/19 °C (day/night) under either long-day (LD: 16 h light/8 h dark) or short-day (SD: 8 h light/16 h dark) photoperiods, with light intensities of 60 µmol photons m^−2^ s^−1^ (LL) or 120 µmol photons m^−2^ s^−1^ (NL) and relative humidity of 70%. Illumination was provided by a combination of Osram HO 80W/840 and HO 80W/830 bulbs. Seeds were stratified at 4 °C for 3 days prior to transfer to growth conditions.

For all the experiments, whole rosettes were harvested in SD at LL light intensity at 40 days after stratification (DAS), at NL at 30 DAS, and in LD at 23 days after stratification. If this set up does not apply in a given method, it is specified in its own section.

### RNA extraction and cDNA synthesis

Total RNA was extracted from rosette leaves of plants grown in SD under LL and NL harvested 40 DAS and 30 DAS, respectively, using the Universal RNA Purification Kit (Roboklon GmbH, Germany) according to the manufacturer’s instructions, and eluted in 30 µl of RNase-free distilled water. The harvest points were one hour before the light was on (− 1), one hour after the light was on (+ 1) and one hour before the light was off (+ 7). RNA concentration and purity were determined using a NanoDrop Nabi™ UV/Vis spectrophotometer (MicroDigital Co. Ltd, South Korea), and RNA integrity was verified by agarose gel electrophoresis. Samples were stored at − 20 °C until further use. To remove potential genomic DNA contamination, RNA samples were treated with the Ambion DNA-free™ DNA Removal Kit (Thermo Fisher Scientific, USA). Subsequently, 2 µg of RNA was reverse-transcribed into complementary DNA (cDNA) using RevertAid Reverse Transcriptase (Thermo Scientific, USA) and Oligo(dT) primers, following the manufacturer’s protocol.

### Reverse-transcription quantitative PCR

Reverse transcription quantitative PCR (RT-qPCR) was conducted using the KAPA SYBR FAST qPCR Kit (KAPA Biosystems Inc., Switzerland) to quantify the expression levels of *MDH1* and *ME2* in cDNA samples derived from the different plant lines. Reactions were performed on a Mastercycler® ep realplex system (Eppendorf, Germany), and cycle threshold (Ct) values were obtained using the accompanying realplex software. Gene expression was normalized to the *ACTIN2* (*ACT2I, At3g18780*) gene, which served as an internal reference.

### Leaf surface area

For the calculation of the leaf surface area and maximum diameter a sample size of *n* = 10 was used from each genotype. The growth process was visually documented throughout the plant development and under NL and LL in SD conditions. Each plant picture was then analyzed using the software ImageJ build 1.50e. The leaf surface areas of all plant lines were determinate using Rosette Tracker image analysis (De Vylder et al. 2012) and were then compared with each other using a two-way ANOVA followed by Tukey’s post-hoc test. Statistical analyses were performed with the software RStudio using the package “multicomp” (version:1.4–29; Hothorn et al. 2008) which compared the leaf surface area of each plant line with each time point (DAS), and the same was applied for the maximum diameter.

### Pulse amplitude modulation fluorometry

PAM (Pulse Amplitude Modulation) measurements were performed using the IMAGING-PAM Maxi version (Walz, Germany) with a PAR (Photosynthetically Active Radiation) of 81 µmol m^−2^ s^−1^. Plants had been dark-adapted for 20 min prior to each measurement. For each plant line, four biological replicates were analyzed, and five measurement points were selected from each replicate. Average values of five points from each biological replicate were used to calculate each photosynthetic parameter. The maximum quantum yield efficiency of PSII (Fv/Fm = [Fm‐F0]/Fm) was taken as a measure of PSII intactness, quantum Yield of PSII (ΦPSII) indicates the amount of solar energy converted to chemical energy to be used by the plant cell, the electron transport rate (ETR) shows the speed of the electrons in the electron transport chain and the non-photochemical quenching (NPQ).

### Transmission electron microscopy

The fourth and fifth leaves from plants grown under SD conditions at 60 µE were excised one hour before the onset of the light period and immediately fixed in a solution containing 75 mM cacodylic acid sodium buffer (Carl Roth GmbH, Germany) pH 7 using HCl, 2.5% glutaraldehyde, 0.1% sucrose, and 2 mM MgSO_4_ for 4–5 h at 4 °C in a desiccator. Following fixation, leaves were washed three times for 15 min each in 100 mM cacodylate buffer. Post-fixation was performed in 2% osmium tetroxide (OsO_4_) for 2.5 h, followed by three 10-min washes in distilled water. Leaves were then dehydrated through a graded acetone series (15%-50%), with three 10-min incubations at each concentration. Subsequently, samples were incubated for 1 h in 75% acetone containing 1% phosphotungstic acid and 1% uranyl acetate, followed by three 15-min incubations in 80%, 90%, and 100% acetone. After dehydration, samples were infiltrated overnight with a 1:3 mixture of acetone and ERL-4206 resin (10% vinyl cyclohexene dioxide). This was followed by 4.5 h in a 1:1 acetone/ERL mixture, 4 h in a 3:1 acetone/ERL mixture, and finally overnight in pure ERL resin at 70 °C.

Once embedded, leaf sections were cut using an ultramicrotome and mounted on copper mesh grids. For contrast enhancement, grids were stained for 5 min in 1% uranyl acetate, followed by 5 min in a solution of lead nitrate [Pb(NO_3_)_2_] and sodium citrate [Na_3_C_6_H_5_O_7_·2H_2_O], and finally treated with 1 M NaOH, with water washes between each step. Prepared grids were examined using a Zeiss EM 10 transmission electron microscope (1978 model). Ultrathin sections were stained with Azur II 1% (w/v), Methylene blue 1% (w/v) dissolved in sodium tetraborate 1% (w/v) and observed using fluorescence stereomicroscope with 40× magnification lens (Leica MZFLIII).

### Determination of the phytohormone levels

Phytohormones were extracted from the whole rosette using 2-phase-extraction with isopropanol/H_2_O/methylene chloride as described previously (Pan et al. 2008; Wewer et al. 2026)*.* Samples were harvested one hour before the light was on and frozen immediately in liquid nitrogen from plants grown under SD in LL and NL conditions. Tissue was ground using mortar and pestle in liquid nitrogen and approximately 50 mg of fresh tissue was used for each extraction. Phytohormone extracts were then analyzed by LC–MS. Phytohormone separation of phytohormones measured in negative ionization mode (*cis*,*trans*-ABA, JA and SA) was done on a C18 XSelect™ HSST3 HPLC column (2.5 µm, 3.0 mm × 150 mm (Waters)) at a flow rate of 0.35 mL/min using a binary gradient, where solvent A was H_2_O + 0.1% (v/v) formic acid, and solvent B was methanol + 0.1% (v/v) formic acid. The gradient was performed as described in (Wewer et al. 2026). Phytohormone separation of phytohormones measured in positive ionization mode (IAA, tZ9G, cZ9G, tZOG and iPR) was done on a C18 Atlantis™ Premier BEH column (2.5 µm, 2.1 mm X 150 mm (Waters)), with the same gradient. Phytohormone analysis by LC–MS was performed on a maXis 4G Q-TOF instrument (Bruker Daltonics), and quantification was based on relation of peak areas to stable isotope-labelled internal standards (I.S.) as described (Wewer et al. 2026). For comparing the different genotypes, statistical analysis was done using using a two-way ANOVA followed by Tukey’s post-hoc test using multcomp package (version:1.4–29; Hothorn et al. 2008) in R studio. *pval* < 0.05 between the genotypes are considered significant differences.

### Determination of C/N ratio, ammonium concentration and metabolite profiling analysis

For the determination of C/N ratio, whole rosettes were harvested one hour before the end of the night (− 1), one hour after (+ 1) and seven hours after (+ 7) the light was switched on from plants growing under LL and under NL in SD. For ammonium quantification, whole rosettes were harvested from plants grown under LL and NL in SD conditions, either 1 h before the onset of light or 1 h after lights-on. For metabolic profile analysis, the whole rosette leaves were harvested at three time points, one hour before the light was on (− 1), one hour after light was on (+ 1) and one hour before the light was off (+ 7/+15) from plants grown under SD-LL, SD-NL and LD-NL and immediately frozen. Total carbon and nitrogen, ammonium content and metabolic profiles measured using gas chromatography-mass spectrometry (GC–MS) were determined according to Martinez et al. (2026). Statistical analyses were performed in RStudio using one-way ANOVA followed by Tukey’s post hoc test implemented in the multcomp package (version:1.4–29; Hothorn et al. 2008). Differences between genotypes were considered statistically significant at *pval* < 0.05 (Suppl. Data 4).

### Isolation of mitochondria and protein concentration

Rosette from 8-week-old plants grown in SD at NL were harvested one hour after the light was turn on (+ 1). The isolation was performed according to an established protocol (Liu et al. 2023). Protein concentration was determinate using the Bradford assay (1976).

### NAD-ME activity assay

NAD-ME activity in isolated mitochondrial fractions was measured following the protocol described by Tronconi et al. (2008). Activity was assessed by monitoring NADH production using a Spark® microplate reader (Tecan) in 96-well UV-Star Microplates (Greiner Bio-One). The standard reaction mixture contained 50 mM MES-NaOH (pH 6.5), 4 mM NAD⁺, 10 mM L-malate, 5 mM dithiothreitol, 10 mM MnCl_2_, and 10 units of MDH from pig heart (Roche, Germany). Enzymatic activity was normalized to total protein content.

### PEPC activity assay

PEPC activity was measured in protein extracts from plants grown under low light (LL) in short-day (SD) conditions. Whole rosettes were harvested 1 h after the onset of light (+ 1 h). Frozen tissue was ground twice for 15 s in a TissueLyser II (QIAGEN, Germany) at a frequency of 30 Hz. Three biological replicates were analyzed per genotype. Protein extraction was performed according to Hüdig et al. (2022), with slight modifications. Shortly, proteins were extracted using 2 µl of extraction buffer (0.1 M HEPES pH 7.8, 0.1 M NaCl, 10 mM MgCl_2_, 0.5% (v/v) Triton X-100, 10 mM DTT, 2 mM PMSF, 0.1% (w/v)) per mg of tissue. After the addition of the buffer samples were vortexed and incubated 20 min on ice. Afterwards, they were centrifugated at 10,000 g 4 °C 10 min. Supernatant was transferred to a pre-chilled tube and used for the PEPC activity determination. Protein concentration was accessed using by Bradford (1976) (Thermo Fischer, Germany). PEPC activity was measured spectrophotometrically at 340 nm by coupling the production of oxaloacetate to the oxidation of NADH from malate dehydrogenase (MDH) at 30 °C in Spark® microplate reader (Tecan). The reaction mixture contained 50 mM HEPES pH 7.8, 10 mM MgCl_2_, 1 mM NaHCO_3_, 6 mM PEP, 0.15 mM NADH, and 10 U/ml MDH from pig heart (Roche, Germany). The crude extract was incubated with the reaction mixture without PEP till the signal at 340 nm was stabilized, after that the substrate was added to start the reaction and the absorbance was monitored for 20 min. Enzymatic activity defined by µmol of NADH consuming per min was normalized by the protein content in each extract.

### Immunoblot analysis

Immunoblot detection of ME1 was performed using mitochondria isolated from *A. thaliana* and a specific antibody raised against *A. thaliana* ME1 (ImmunoGlob GmbH, Germany). Recombinant *A. thaliana* ME1 was used as a positive control (Tronconi et al. 2008). Immunoblot detection of PEPC1 was performed using total protein extracts from rosettes and an antibody against *A. thaliana* PEPC1 (AS16 4110, Agrisera).

Denaturing polyacrylamide gel electrophoresis (SDS-PAGE) was carried out on 14% (w/v) polyacrylamide gels according to Laemmli (1970). Proteins were then transferred onto a nitrocellulose membrane for 1.5 h at 133 mA for ME1 and 200 mA for PEPC1 using a semi dry from Biorad system. Membranes were blocked for 1 h at room temperature in TBS (20 mM Tris–HCl, pH 7.5, 150 mM NaCl) supplemented with 5% (w/v) milk powder. After blocking, the membrane was washed three times for 10 min using TBS-T (0.1% (w/v) Tween 20 in TBS). For ME1 detection, the membrane was incubated overnight at 37 °C with 0.5 µg/µl of the primary antibody diluted in TBS containing 5% (w/v) milk powder. For PEPC1 detection, the membrane was incubated overnight at 4 °C with the primary antibody diluted 1:1000 in TBS with 2% (w/v) milk powder. Following primary antibody incubation, membranes were washed three times with TBS-T, following incubation with goat anti-rabbit HRP-conjugated secondary antibody, diluted 1:5000 for ME1 detection (Merck, Germany) or 1:40,000 for PEPC1 detection (AS09602, Agrisera). After three additional washes with TBS-T, signal detection was performed by incubating the membranes for 5 min in a 1:1 mixture of ECL (enhanced chemiluminescence) substrate solution (Western Blot kit, Bio-Rad). Chemiluminescence signals were recorded using an Azure 300 imaging system (Azure Biosystems, USA) for ME1 and a ChemiDoc MP Imaging System (Bio-Rad) for PEPC1.

### Oxford nanopore technology sequencing

High molecular weight DNA was extracted from young *A. thaliana* plants using a modified CTAB-based method (Siadjeu et al. 2020). The Short Read Eliminator kit (Pacific Biosciences) was used to deplete short DNA fragments from the samples. Samples were prepared for nanopore sequencing following the SQK-LSK114 protocol starting with 1 µg of DNA. Sequencing was performed on R10.4.1 flow cells on a MinION Mk1B. POD5 files were subjected to basecalling with dorado v1.0.2 (https://github.com/nanoporetech/dorado) using the high accuracy model dna_r10.4.1_e8.2_400bps_hac@v5.2.0. Reads were aligned to the TAIR10 reference genome sequence with minimap v2.27-r1193 (Li 2018) using default parameters for nanopore reads. Samtools v1.19.2 (Li et al. 2009) was used to convert SAM into BAM files for visualization in Integrative Genomics Viewer v2.7.2 (Robinson et al. 2011). Bedtools (Quinlan and Hall 2010) was utilized to infer coverage for each genomic position (Pucker and Brockington 2018). Loreta v1 (Pucker et al. 2021) was deployed for the investigation of T-DNA insertions and structural rearrangements. The sequences of pAC161 (AJ537514) and pCSA110 (McElver et al. 2001) served as bait for the discovery of T-DNA sequences. A dedicated Python script was applied for the genome-wide coverage analysis (available viaGitHub: https://github.com/bpucker/GKseq2).

### RNA seq analysis

For RNA extraction, rosette leaves were harvested one hour before (-1) and after (+1) the light was switched on from plants growing in SD under LL and NL and LD under NL. Total RNA was extracted with the SV Total RNA Isolation System (Promega) and genomic DNA was eliminated by treating the samples with DNase I using DNA-free™ Kit (Invitrogen, Thermo Fischer), 1 µl of rDNase I was used per sample incubating at 37 °C for 30 min. Total RNA was quantified by the Qubit RNA HS Assay (Thermo Fisher Scientific, MA, USA). The quality was assessed by capillary electrophoresis using the Fragment Analyzer and the ‘Total RNA Standard Sensitivity Assay’ (Agilent Technologies, Inc. Santa Clara, CA, USA). Three biological replicates per genotype and condition with RNA Quality Numbers (RQN) mean > 8.2 were analyzed. The library preparation was performed according to the manufacturer’s protocol using the ‘VAHTS™ Stranded mRNA-Seq Library Prep Kit’ for Illumina®. Shortly, 400 ng total RNA were used as input for mRNA capturing, fragmentation, the synthesis of cDNA, adapter ligation and library amplification. Bead purified libraries were normalized and finally sequenced on the NextSeq2000 system (Illumina Inc. San Diego, CA, USA) with a read setup of 1×100 bp. The BCL Convert Tool (version 3.8.4) was used to convert the BCL files to FASTQ files as well for adapter trimming and demultiplexing.

Data analyses on FASTQ files were conducted with CLC Genomics Workbench (version 23.0.2, Qiagen, Venlo, Netherlands). The reads of all probes were adapter trimmed (Illumina TruSeq) and quality trimmed (using the default parameters: bases below Q13 were trimmed from the end of the reads, ambiguous nucleotides maximal 2). Mapping was done against the *A. thaliana* (TAIR10.54) (July 20, 2022) genome sequence. The differential expression analysis was performed using the DEseq2 package (Love et al. 2014) from BiocManager in the software RStudio, gene with less than five reads were depreciated. The Resulting *p* values calculated by Wald test were corrected for multiple testing using Benjamini and Hochberg method (BH-adjusted *p* values). An adjusted *p* value of ≤ 0.05 was considered significant.

### Shotgun proteomic LC–MS analysis

Proteomic analysis was performed on plants grown under SD conditions at light intensities of LL and NL. Whole rosettes were harvested 1.5 h after the onset of light. Five biological replicates per genotype were collected. Protein extraction and digestion was carried out using a single-pot, solid-phase-enhanced sample preparation (SP3) protocol (Hughes et al. 2019), which was adapted to meet challenges inherent to plant samples (Mikulášek et al. 2021). Each extract was treated with 2 µg of pre-activated, sequencing grade Trypsin (V5111, Promega). Concentrations of extracted tryptic peptides were determined in a plate reader (Multiskan Sky, Thermo Fischer Scientific) using the Pierce Quantitative Colorimetric Peptide Assay Kit (Thermo Fisher Scientific, Dreieich, Germany) following the manufacturer’s instructions. Peptides were dried using a vacuum concentrator and resuspended in 0.1% (v/v) formic acid (FA) to yield a peptide concentration of 10 ng µl^−1^. Twenty microliters of the suspension were loaded on Evotips (Evosep) according to the manufacturer’s instructions for desalting and subsequent separation using an EvoSep One HPLC (Evosep). Peptides bound to the Evotip resin were eluted using the Whisper Zoom 40 SPD method and separated in a 15 cm Aurora Elite CSI column (inner diameter 75 µm, particle size 1.7 µm, pore size 120 Å, IonOpticks, Fitzroy, Australia) which was kept at a temperature of 50 °C. Eluting peptides were transferred to a timsTOF Pro mass spectrometer (Bruker, Germany) and analyzed using the pre-installed ‘DDA PASEF-short_gradient_0.5sec_cycletime’ method.

Acquired spectra were queried against an in-house modified TAIR10 database using the MaxQuant software (Cox and Mann 2008) version 2.6.3.0. Default parameters were used, and the ‘iBAQ’ (Schwanhäusser et al. 2011) and ‘LFQ’ functions were additionally enabled for quantification of identified protein groups.

The resulting ‘proteingroups.txt’ file was complemented with predicted subcellular protein localization using the SUBAcon algorithm (Hooper et al. 2014) and Mapman annotation (Thimm et al. 2004). Clasified table was separated in two tables: plastid-protein groups and non-plastid-protein groups filtered by SUBAcon. These tables were submitted to statistical analysis by means of the Perseus software (Tyanova et al. 2016) version 2.1.2.0 in the form of two‐sample t tests (*p* < 0.05). For this, protein groups matching ‘only identified by site’, ‘potential contaminant’ and ‘reverse’ were removed before LFQ values were log_2_-transformed. Protein groups with less than three values in each sample group were removed and missing values were imputed from normal distribution using a width of 0.3 and a down-shift of 1.8. A width normalization was performed volcano plots were produced using default parameters (FDR, 0.05; S_0_, 0.1).

## Supplementary Information

Below is the link to the electronic supplementary material.

Supplementary file1 (DOCX 1167 KB)

Supplementary file2 (XLSX 90 KB)

Supplementary file3 (XLSX 4598 KB)

Supplementary file4 (XLSX 80 KB)

Supplementary file5 (XLSX 54 KB)

## Data Availability

All MS raw files acquired in this study, along with the corresponding MaxQuant search results can be downloaded from the FTP server ftp://massive-ftp.ucsd.edu/v12/MSV000101094/. Or on the MassIVE website using the MassIVE identifier: MSV000101094.
