# Supplementary material for: A hidden T-DNA-linked inversion-duplication is associated with a light-dependent Arabidopsis mutant phenotype
Source: Plant Cell Rep. 2026 Nov 3;45(11):350. doi: 10.1007/s00299-026-04030-1 (PMC13731719; doi:10.1007/s00299-026-04030-1)
Supplement: Supplementary file 1 [file 299_2026_4030_MOESM1_ESM.docx]

**Supplementary Figures**

**Supplementary Figure 1. T-DNA insertion lines of *A. thaliana* mitochondrial *ME2* and *MDH1*. A)** Gene models of *ME2* and *MDH1* indicating the confirmed T-DNA insertion sites. Black arrows indicate locations of primer binding sites used for zygosity analysis and RT-qPCR (Suppl. Table 2). Rectangles represent exons and connecting lines represent introns. **B)** Relative expression levels of *ME2* and *MDH1* in rosette leaves of 40-day-old Col-0 and double insertional mutants grown in SD and LL and harvested 1 h before onset of the light. Data represent means ± SD (n = 3). Transcript abundances in *mdh1.1 me1 me2.1/2* are published in Martinez et al. (2026). Significance relative to Col-0 was assessed by Welch's t-test (*p* < 0.05, *; *p* < 0.01, **; *p* < 0.001, ***; *p* < 0.0001, ****).

**Supplementary Figure 2. Proposed inheritance scenario for the structural variant linked to the mdh1.1 locus.** The mdh1.1 parental population likely segregated for alternative **MDH1-linked configurations** that carried the same T-DNA insertion but differed in the presence of the adjacent inverted duplication. Because plants were selected under LD-NL conditions, where the duplication-associated phenotype is not apparent, these configurations could not be distinguished visually. Selection therefore depended only on the desired T-DNA genotypes. During line generation, the duplicated configuration was retained by chance in the mdh1. 1me2 double mutants, whereas the non-duplicated configuration was retained by chance in the mdh1.1 me1 me2 triple mutant. Red boxes indicate the genotypes propagated in each crossing scheme. Symbol lengths are not drawn to scale. N, northern end of chromosome; S, southern end of chromosome.

**Supplementary Figure 3. Analysis of the differential expressed genes from the duplicated region under the light-dark transition in SD.** Venn diagram (created using Venny 2.1) showing common and condition-unique high abundance transcripts from the duplicated region in *mdh1.1 me2.1* at LL and NL and -1 and +1 (high abundance transcripts: log_2_FC > 1; n = 3 per genotype and condition). LL: low light; NL: normal light. Supplementary Data 1.

**Supplementary Figure 4. Differentially expressed genes between *mdh1.1 me2.1* and *mdh1.1 me1 me2.1*. A)** Number of differentially expressed genes under SD-LL and SD-NL one hour before (-1) and one hour after (+1) the lights on. **B)** Gen ontology (GO) enrichment using STRING database with up- and down-DEGs one hour after the light is on (+1) under SD-LL. Gene count indicates how many proteins in your network are annotated with a particular term. Signal is defined as a weighted harmonic mean between the observed/expected ratio and -log(FDR). FDR (False Discovery Rate) tends to emphasize larger terms due to their potential for achieving lower p-values, while the observed/expected ratio highlights smaller terms, which have a high foreground to background ratio but cannot achieve low FDR values due to their size. The signal measure seeks to balance both metrics for more intuitive ordering of enriched terms. False Discovery Rate (FDR), this measure describes how significant the enrichment is. Shown are p-values corrected for multiple testing within each category using the Benjamini–Hochberg procedure. LL: low light; NL: normal light. Supplementary Data 2.

**Supplementary Figure 5. C/N balance and ammonium accumulation.** Carbon-to-nitrogen ratio (C/N), calculated as total carbon divided by total nitrogen, total carbon (%C) and total nitrogen (%N) (percent of dry weight) were quantified in rosette leaf samples harvested 1 h before lights-on (-1 h), 1 h after lights-on (+1 h), and 1 h before lights-off (+7 h). Total leaf NH_4_^+^ concentrations were measured at -1 h and +1 h. Plants were grown under short-day (SD) conditions at low light (LL; **A**) or normal light (NL; **B**). Data represent means ± SD from 3-4 independent biological replicates per genotype. Significance relative to Col-0 was determined using Welch’s t-test (p < 0.05, *; p < 0.01, **; p < 0.001, ***; p < 0.0001, ****).

**Supplementary Figure 6. Relative metabolite abundance. Rosette of** *mdh1.1 me2.1* **and Col-0 were harvested 1 h before lights-on (-1 h), 1 h after lights-on (+1 h) and 1 hour before lights-off (+7/15) grown in SD at LL and NL, and at NL in LD. Statistical analyses of n=6 independent biological replicates were performed for each time point using** ANOVA followed by Tukey’s HSD**. Asterisks show metabolites that differ significantly at p-val < 0.05 from the wild type. Supplementary Data 4.**

**Supplementary Figure 7. Relative metabolite abundance. Rosette of** mdh1.1 me2.2 **and Col-0 were harvested 1 h before lights-on (-1 h), 1 h after lights-on (+1 h) and 1 hour before lights-off (+7/15) grown in SD at LL and NL, and at NL in LD. Statistical analyses of n=6 independent biological replicates were performed for each time point using** ANOVA followed by Tukey’s HSD**. Asterisks show metabolites that differ significantly at pval < 0.05 from the wild type. Supplementary Data 4.**

**Supplementary Figure 8. Hypothetical metabolic model linking enhanced PEPC1 activity with MDH1 function.** The scheme illustrates a possible metabolic relationship between increased phosphoenolpyruvate carboxylase 1 (PEPC1) activity and mitochondrial MDH1 function. Enhanced PEPC1 activity may increase cytosolic oxaloacetate (OAA) production from phosphoenolpyruvate (PEP), while impaired MDH1 activity may alter mitochondrial malate (Mal)/OAA metabolism and metabolite exchange between cellular compartments. These changes could potentially converge on OAA availability and thereby influence downstream carbon and nitrogen metabolism, including OAA-derived amino acid biosynthesis. The proposed connection between PEPC1 and MDH1 is hypothetical. DiT1, chloroplastic dicarboxylate transporter 1; DIC, mitochondrial dicarboxylate carrier.

**Supplementary Figure 9. Phytohormone profiling.** Whole rosettes were harvested 1 h before lights-on (-1 h) from plants grown under short-day (SD) conditions at normal light (NL) or low light (LL) to quantify phytohormone levels in mdh1.1 me2.1, mdh1.1 me1 me2.1 and Col-0. For each condition, 6-7 independent biological replicates were analyzed, and hormone abundance was normalized to fresh weight. Statistical analyses were performed separately for each light intensity using one-way ANOVA followed by Tukey’s post-hoc test; different letters indicate significant differences (*p* < 0.05). NL, normal light (120 µmol photons m^-2^ s^-1^); LL: low light (60 µmol photons m^-2^ s^-1^); LD, long-day; SD, short-day; IAA: Indole-3-acetic acid; SA: salicylic acid; JA: Jasmonic acid; c,t-ABA: Abscisic acid; tZ9G: trans-Zeatin-9-glucoside; cZ9G: cis-zeatin-9-glucoside; tZOG: trans-Zeatin O-glucoside; iPR: N6-isopentenyladenosine.

**Supplementary Tables (Martinez et al., 2026)**

**Supplementary Table 1.** Information on the T-DNA insertion lines used in the work. s. a., see above; ko. knock out; kd, knock down.

| **Mutant (Type)** | **Gene** | **Gene locus** | **Insertion line** | **Reference** | **Phenotype**  **SD-LL** |
| --- | --- | --- | --- | --- | --- |
| *mdh1.1* (ko) | *MDH1* | *At1g53240* | GK-097C10 | (Tomaz et al., 2010) | no |
| *mdh1.2* (ko) | *MDH1* | *At1g53240* | GABI_540F04 |  | no |
| *me1* (ko) | *ME1* | *At2g13560* | Sail-374-A02 | (Tronconi et al., 2008) | no |
| *me2.1* (ko) | *ME2* | *At4g00570* | Sail-291-C05 |  | no |
| *me2.2* (kd) | *ME2* | *At4g00570* | SALK_131720 |  | no |
| *mdh1.1 me1 me2.1* | s.a. | s.a. | s.a. | (Martinez et al., 2026) | yes |
| *mdh1.1 me1 me2.2* | s.a. | s.a. | s.a. |  | yes |
| *mdh1.1 me2.1* | s.a. | s.a. | s.a. | this work | yes |
| *mdh1.1 me2.2* | s.a. | s.a. | s.a. |  | yes |
| *mdh1.2 me2.1* | s.a. | s.a. | s.a. | this work | no |
| *mdh1.2 me2.2* | s.a. | s.a. | s.a. |  | no |

Supplementary Table 2. List of primers used in this study. s. a., see above.

| **Name** | **Sequence (5'- 3')** | **Gene/T-DNA Insertion** | **AGIs** |
| --- | --- | --- | --- |
| me2-Fw1 | GACCTGTGTACAGCAATGTGATCG | *ME2* | *At4g00570* |
| me2-Rv1 | CCTGGACATCATCATTGAACATGC |  |  |
| mdh1-Fw1 | TCCGATCTTCTGCCTCC | *MDH1* | *At1g53240* |
| mdh1-Rv1 | CAACTGGGACATTTGCCTT |  |  |
| act2-Fw1 | TAACTCTCCCGCTATGTATGTCGC | *ACT2* | *At3g18780* |
| act2-Rv1 | GAAGCAAGAATGGAACCACCG |  |  |
| Sail | TAGCATCTGAATTTCATAACCAATCTCGATACAC | *ME2.1* | s. a. |
| SALK | TGGTTCACGTAGTGGGCCATCG | *ME2.2* | s. a. |
| GABI | ATATTGACCATCATACTCATTGC | *MDH1.1/.2* | s. a. |
| me2-Fw2 | TTGGTGTAACTAAGATGGCAGT | *ME2* | s. a. |
| me2-Rv2 | CGTATCTCAGCTGGATTTTTGG |  |  |
| mdh1-Fw2 | TGTCCTCTCCGTGGTGACAAGACC | *MDH1* | s. a. |
| mdh1-Rv2 | TCCGATCTTCTGCCTCC |  |  |
| act2-Fw2 | CTTGCACCAAGCAGCATGAA | *ACT2* | s. a. |
| act2-Rv2 | CCGATCCAGACACTGTACTTCCTT |  |  |

**Supplementary Table 3.** List of *cis*-elements identified in the promotor region of *PEPC1* by PlantCARE.

| Motif | Position upstream of the transcript start site (bp) | Strand | Sequence | Function |
| --- | --- | --- | --- | --- |
| ACE | -454 | - | GACACGTATG | cis-acting element involved in light responsiveness |
|  | -830 | + | CTAACGTATT |  |
| Box 4 | -166 | + | ATTAAT | part of a conserved DNA module involved in light responsiveness |
|  | -588 | + | ATTAAT |  |
| ATC-motif | 1261 | + | AGTAATCT | part of a conserved DNA module involved in light responsiveness |
| G-box | 456 | + | TACGTG | cis-acting regulatory element involved in light responsiveness |
| GT1-motif | -707 | - | GGTTAA | light responsive element |
|  | -1561 | - | GGTTAA |  |
|  | -1560 | - | GGTTAAT |  |
|  | -1622 | - | GGTTAA |  |
